# A novel TF molecular switch-mechanism found in two contrasting ecotypes of a psammophyte, *Agriophyllum squarrosum*, in regulating transcriptional drought memory

**DOI:** 10.1186/s12870-023-04154-6

**Published:** 2023-03-30

**Authors:** Tingzhou Fang, Chaoju Qian, Bachir Goudia Daoura, Xia Yan, Xingke Fan, Pengshu Zhao, Yuqiu Liao, Liang Shi, Yuxiao Chang, Xiao-Fei Ma

**Affiliations:** 1 Key Laboratory of Ecological Safety and Sustainable Development in Arid Lands, Northwest Institute of Eco-Environment and Resources, Chinese Academy of Sciences, Lanzhou, Gansu 730000 China; 2 College of Resources and Environment, University of Chinese Academy of Sciences, Beijing, 100049 China; 3 Department of Biology, Faculty of Sciences and Technology, Dan Dicko Dankoulodo University, POBox 465, Maradi, Niger; 4 Key Laboratory of Eco-hydrology of Inland River Basin, Northwest Institute of Eco- Environment and Resources, Chinese Academy of Sciences, Lanzhou, Gansu, 730000 China; 5 Agricultural Genomics Institute at Shenzhen, Chinese Academy of Agricultural Science, Shenzhen, 518000 China

**Keywords:** Drought memory, *Agriophyllum squarrosum*, Molecular switch, Comparative transcriptomics, Psammophytes, Local adaptation

## Abstract

**Background:**

Prior drought stress may change plants response patterns and subsequently increase their tolerance to the same condition, which can be referred to as “drought memory” and proved essential for plants well-being. However, the mechanism of transcriptional drought memory in psammophytes remains unclear. *Agriophyllum squarrosum*, a pioneer species on mobile dunes, is widely spread in Northern China’s vast desert areas with outstanding ability of water use efficiency. Here we conducted dehydration-rehydration treatment on *A. squarrosum* semi-arid land ecotype AEX and arid land ecotype WW to dissect the drought memory mechanism of *A. squarrosum*, and to determine the discrepancy in drought memory of two contrasting ecotypes that had long adapted to water heterogeneity.

**Result:**

Physiological traits monitoring unveiled the stronger ability and longer duration in drought memory of WW than that of AEX. A total of 1,642 and 1,339 drought memory genes (DMGs) were identified in ecotype AEX and WW, respectively. Furthermore, shared DMGs among *A. squarrosum* and the previously studied species depicted that drought memory commonalities in higher plants embraced pathways like primary and secondary metabolisms; while drought memory characteristics in *A. squarrosum* were mainly related to response to heat, high light intensity, hydrogen peroxide, and dehydration, which might be due to local adaptation to desert circumstances. Heat shock proteins (HSPs) occupied the center of the protein-protein interaction (PPI) network in drought memory transcription factors (TF), thus playing a key regulatory role in *A. squarrosum* drought memory. Co-expression analysis of drought memory TFs and DMGs uncovered a novel regulating module, whereby pairs of TFs might function as molecular switches in regulating DMG transforming between high and low expression levels, thus promoting drought memory reset.

**Conclusion:**

Based on the co-expression analysis, protein-protein interaction prediction, and drought memory metabolic network construction, a novel regulatory module of transcriptional drought memory in *A. squarrosum* was hypothesized here, whereby recurrent drought signal is activated by primary TF switches, then amplified by secondary amplifiers, and thus regulates downstream complicated metabolic networks. The present research provided valuable molecular resources on plants’ stress-resistance basis and shed light on drought memory in *A. squarrosum*.

**Supplementary Information:**

The online version contains supplementary material available at 10.1186/s12870-023-04154-6.

## Introduction

Due to their sessile properties, plants can adjust their survival strategies in a series of multi-level regulatory patterns involving morphological alterations, cell physiological regulation, and gene expression differences to cope with environmental factors [1]. It has been reported that prior stress may change plant response patterns, and subsequently increase its tolerance to the same stress (in some cases, it may also be more sensitive), commonly referred to as “domestication”, “exercise” or “stress memory” [2, 3]. Exploration of the mechanisms of plant memory helps dissect the fundamental issue of stress resistance and has attracted wide interest. The hypothesis proposed by Thellier and Luttge suggests two possible plant memory mechanisms: a linear pathway starting with signal reception, amplifying by effectors, and eventually forming memory, which can be described in the terms “learning”, “habit”, or “training”; or memory storage and recalling mechanism based on complex networks with highly integrated and feedback effects [4].

Drought is one of the most frequent and severe environmental stresses [5]. Water deficit jeopardizes plant development by reducing photosynthetic capacity, root vitality, water utilization, and leads to accumulated reactive oxygen species (ROS), which may cause permanent damage and subsequent severe loss of productivity to plants [6, 7]. Water deficit repeats frequently throughout the plant life history in wild, thus, as an integral part and one of the measurements of plants drought resistant system, strong drought memory, instead of mere actively drought response, is essential for enhancing plant drought tolerance. Progress has been made in plant drought memory research. Plant drought memory may be driven by changes in key signaling metabolites or transcription factors (TFs) on physiological or molecular levels, and may also involve chromatin states altering such as histone tail modifications, DNA methylation, or RNA polymerase II stalling, namely, changes at the epigenetic level [8]. On the transcriptional level, drought memory genes (DMGs) are defined as those drought-responsive genes with significantly transcription level and speed changes between the first and the subsequent water deficit, histone trimethylation modification (H3K4me3) and stalled RNA polymerase II (Ser5P Pol II) were proved to be potential epigenetics modification markers in drought memory [9]. Furthermore, TFs might also function as regulators in drought memory. For instance, members of the TF family of ABF, MYB, WRKY, NAC, ERF, etc. were identified as DMGs [10–14]. Additionally, microRNA was speculated to play important roles in drought memory from their regulatory functions in thermomemory [11]. According to their expression profiles in *Arabidopsis thaliana* and *Zay mays*, DMGs were sorted into four distinct types, [+/+], [+/−], [−/−], and [−/+], whose function could be summarized as four patterns that increase plant resistance and viability, including protection and repair enhance, growth and resistance trade-off, homeostasis readjustment, and crosstalk between multiple stresses [15, 16]. In general, dynamic changes in the expression level of DMG resulted in an over-all physiological alternation, including the following aspects: (1) photosynthesis and energy metabolism [17–19]; (2) Osmotic readjustment and water status alter [10, 14, 20–23]; (3) Cellular detoxification system [13, 14, 24, 25]; (4) Phytohormone signaling transduction [26–28]; (5) Memory resetting. Sustaining stress memory states (morphological acclimation, physiological changes, molecular and metabolic alterations) endows plants with a stronger ability of stress-defending yet has negative impacts on plant growth. Hence, it can be favorable to learn to reset (a.k.a. stress forgetfulness), which has been proposed as the main plant strategy to fine-tune growth in fluctuating and unpredictable environmental conditions [8]. Nevertheless, information on plant drought memory resetting remains largely unknown. These findings unveiled that adaptation to recurring drought stress involved the differential expression of DMGs, thus leading to the reprogramming and collaboration of multiple metabolism pathways that coordinately render the plants stronger resistance when encountering subsequent water scarcity.

On the other hand, as a consequence of the in situ adaptation to heterogeneous water conditions, plants have adopted diverse drought memory strategies. Psammophytes have evolved life-history countermeasures adapted to their harsh in situ environments with extremes of heat, drought, and infertility, and thus have accumulated numerous valuable stress-resistant genetic resources [29]. In regard of drought memory, it might be speculated that psammophytes may activate a more refined molecular regulation. However, how desert plants respond to repeating drought stresses remains largely unknown. *Agriophyllum squarrosum* (L.) Moq. is a pioneer annual plant of the Chenopodiaceae family, and is widely distributed in the mobile sand dunes of all deserts and sandy lands across the Asian interior [30]. In our previous research, *A. squarrosum* can survive at a soil moisture content of only about 3% (unpublished data). Additionally, *A. squarrosum* is of great nutritional and medicinal qualities [31, 32], endowing the plant with highly domestication potential as a promising pseudocereal in sandy land. To dissect the molecular memory mechanism of *A. squarrosum* responding to recurring drought, and to determine the discrepancy in drought memory of the two ecotypes that had long adapted to water heterogeneity in their original habitats, here we conducted physiological traits monitoring and comprehensive transcriptomic analysis on two contrasting ecotypes of *A. squarrosum* under three rounds of dehydration-rehydration treatments. A comparison between previously studied species and *A. squarrosum* was introduced in our research to assess the peculiar drought memory nature of *A. squarrosum*. Ultimately, we offered a possible reticulum module of *A. squarrosum* drought memory mechanism, which provided evidence for the hypothesis of complex memory storage and recalling networks in plants. Our findings shed light on psammophytes’ drought memory mechanisms, which can not only provide a theoretical basis for the domestication of pseudocereals to cultivate high-yield and strong adaptive varieties but also commit to the development of an ecological civilization in the vast sandy land.

## Results

### Physiological discrepancies between the two ecotypes of ***A. squarrosum*** during recurring drought treatment

**Fig. 1:**
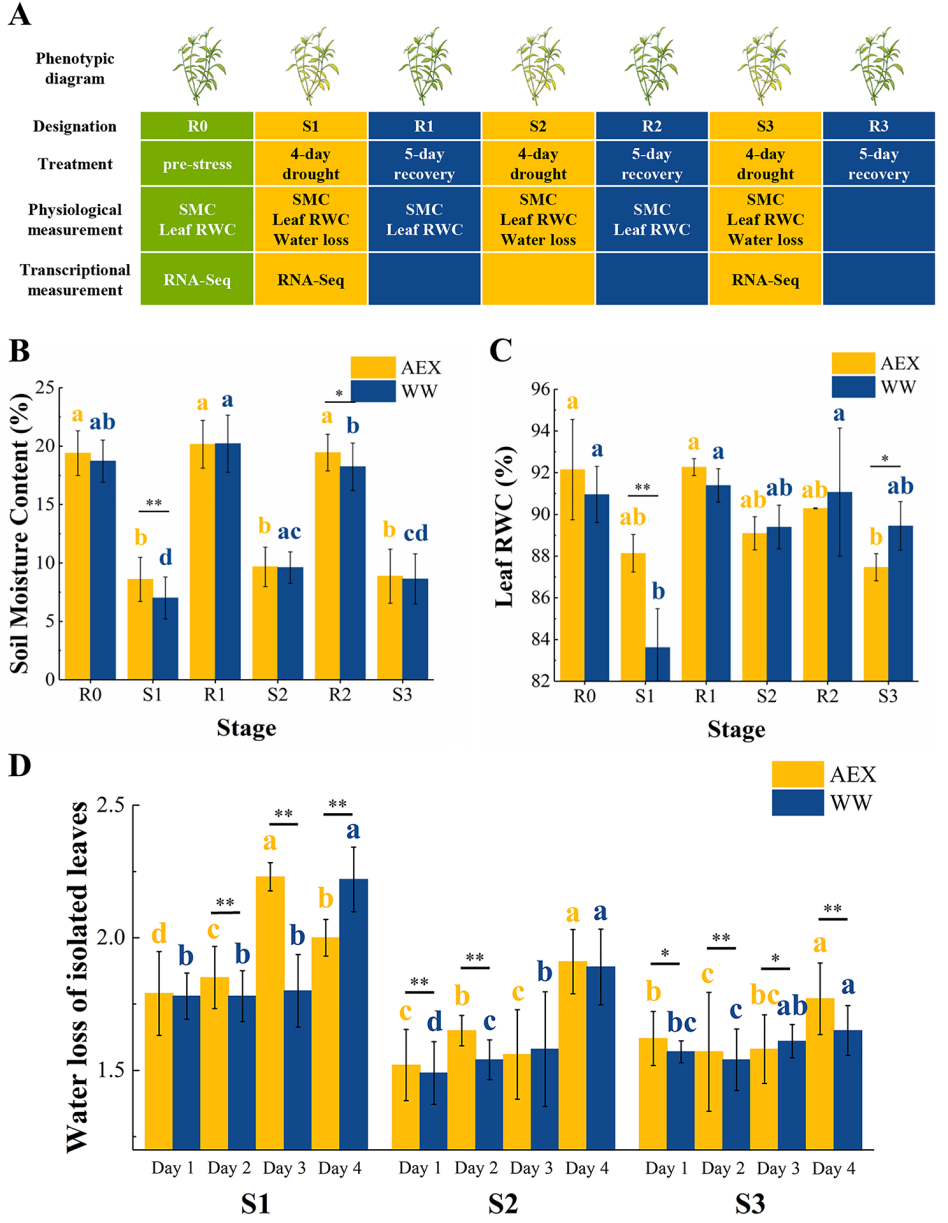
**(a)** Experiment design, physiological index in drought memory trial of **(b)** SMC, **(c)** leaf RWC, and **(d)** water loss of isolated leaves, respectively. R0, control; S1-3, the first, the second, and the third round of dehydration treatments, respectively; R1-3, the corresponding water recovery after S1-3, respectively. SMC, soil moisture content; RWC, relative water content. Indexes were shown in mean ± SD, n = 10. Within the same group (ecotype), the same superscripts denoted no significant difference (P > 0.05); different superscripts denoted significant difference (P < 0.05). Asterisks on shoulder lines denote statistically significant differences: *, p < 0.05; **, p < 0.01, between groups (ecotypes)

The variation of soil moisture content between the two ecotypes showed no obvious discrepancy, providing an equivalent water condition throughout the trial (Fig. 1b). In general, drought resistance of plants is negatively correlated with the water loss of isolated leaves, which is one of the indicators of water retention capacity [33]. Leaf RWC in AEX and WW both fluctuated along with the water condition range, and the degree of RWC reduction from the reappeared drought treatment decreased compared to the first dehydration (Fig. 1c). Nevertheless, closer inspection of the compared RWC from each stage between two ecotypes showed interesting differences. In AEX, a consecutive decline of RWC in R2 and S3 was observed, indicating an attenuation in drought memory function. In contrast, no such decline was found in WW ecotype. Though significant RWC decline (p-value < 0.01) was observed in WW stage S1, RWC in R1, S2, R2, and S3 remained surprisingly at the same level, which was higher than that in AEX. Moreover, as the different variation trends in leaf RWC between the two ecotypes showed the duration and/or intensity discrepancy of stress memory, water loss in S1 was higher than that in S2 and S3 in both ecotypes (though not significantly; Fig. 1d**)**, while the variation of S3 water loss was leveling out without an obvious peak, indicating the fact that drought resistance was enhanced, or ‘trained’, due to the first dehydration treatment. Comparing the two ecotypes in S1, the peak value appeared on day 4 in WW and day 3 in AEX, which means the former one has a relatively higher water retention capacity, and thus a stronger intensity of initial drought resistance. However, in stage S2, this contrast seemed to disappear. The capacity of drought resistance in AEX was advanced in S2, depicted as a delayed peak of water loss on day 4.

### Drought memory genes (DMGs) classification in ***A. squarrosum***

Twelve samples were used for sequencing. After removal of sequences with low quality, poly-N, and adaptors, a total of 86.8 Gb clean reads were generated, with an average of 7.23 Gb reads per sample and a Q30 average quality score of 94.2% (Additional File 1). These high-quality trimmed clean reads were *de novo* assembled into contigs and then a joint transcript of 52,925 unigenes. Annotation of all transcripts in *A. squarrosum* generated 32,488 annotated unigenes (about 61.4% of the whole transcript) in at least one of the seven databases (Additional File 1). GO enrichment of the whole transcript illustrated that unigenes were mainly focused on cell, cell part, and organelle in CC (Cellular Component), binding and catalytic activity in MF (Molecular Function), cellular process, metabolic process, and response to stimulus in BP (Biological Process; Additional File 2a). The following three COG terms were found the most abundant in unigenes: amino acid transport and metabolism, cell wall/membrane/envelop biogenesis, and cytoskeleton (Additional File 2b). The annotated transcripts shared high similarity with *Chenopodium quinoa* (10,047, 30.9%), followed by *Spinacia oleracea* (7,294, 22.5%) and *Beta vulgaris* (4,405, 13.6%; Additional File 2c). Unigenes without annotation were designated as novel genes in *A. squarrosum*.

The transcripts of AEX and WW embraced 43,170 and 41,458 genes, respectively, with varied expression profiles (Additional File 3, 4a). GO enrichment analysis was conducted in each DEG set within the same ecotype. The main function of genes responding to drought stress in both AEX and WW were cell and cell part, in CC, binding and catalytic activity in MF, cellular and metabolic process in BP (Additional File 4b-e). PCA of all the samples divided the two ecotypes along dim1, and different treatments were separated along dim2 (Additional File 5a); while correlation coefficient depicted great repeatability of biological duplicates within each sample (Additional File 5b). Thoroughly investigation into transcripts data unveiled that 5,379 (AEX, 12.5%) and 4,436 (WW, 10.7%) genes were significantly up/down-regulated in S1 versus R0, respectively, representing the drought-responsive genes in *A. squarrosum*. About 10% of *A. squarrosum* genes were identified as drought-responsive genes, which was much more than that in *A. thaliana* (3.3%) and *Z. mays* (5.2%), while half the amount of *P. virgatum* (19.6%). DMGs were identified and categorized into four subgroups based on their expression profiles (Additional File 6, 7). Within both two ecotypes of *A. squarrosum*, about 32% of the genes were identified as DMGs. This proportion is quite similar to that of *A. thaliana* (29.8%) but less than that of *Z. mays* (39.6%) and *P. virgatum* (47.3%). Though the percentage of four types of DMGs varied among these species, subgroup [+/−] in *A. squarrosum* dominated all DMGs, which was in accordance with other studied plants (Table 1). In ecotype AEX, DMG subgroups [+/+] and [−/−], which showed continuously amplified expression along the dehydration stress, contained 138 and 128 genes, respectively. Subgroups [+/−] and [−/+], with reverse expression patterns in S1 and S3, each were comprised of 860 and 650 genes. Meanwhile in ecotype WW, gene distribution in four subgroups [+/+], [−/−], [+/−], and [−/+] was 115, 165, 539, and 520, respectively. The proportion of late-response genes in *A. squarrosum* (3.1% in AEX and 2.6% in WW, respectively), however, was smaller than any other studied species, e.g., *P. virgatum*, 11.9%, *Z. mays*, 7.4%, and *A. thaliana*, 4.1% (Table 1).

### The distribution of DMGs illustrated drought memory commonalities in higher plants and characteristics in ***A. squarrosum***

**Fig. 2:**
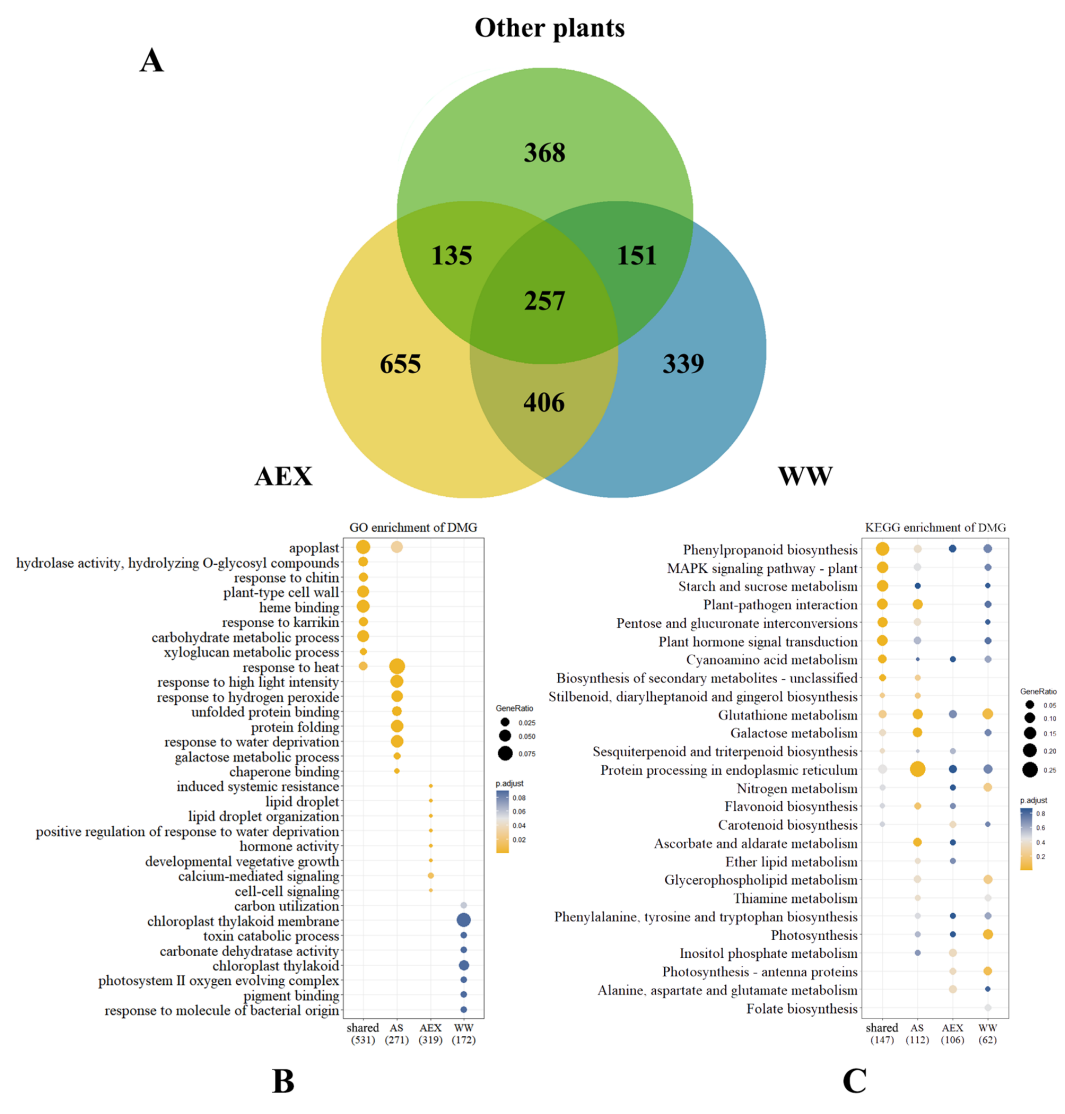
Distribution **(a)**, GO **(b)**, and KEGG **(c)** enrichment of DMG OGs among *A. squarrosum* and other plants. DMGs in the three previously studied species without homologous in *A. squarrosum* were combined into one category and labeled as ‘other plants’. The intersection of “other plants”, “AEX”, and “WW” resulted in 257 shared DMG OGs; the intersection of “AEX” and “WW” while “other plants” excluded resulted in 406 OGs that peculiar in *A. squarrosum*, and named as AS DMG; the 655 and 339 OGs unique in the two ecotypes were designated as AEX DMG and WW DMG, respectively. Figure 2b and c presented the biofunction prediction of the four non-overlapping DMG hierarchies, respectively. Noted that enrichment analyses were performed based on shared, AS, AEX, and WW DMG hierarchies, instead of all the DMG in each species. Thus, “extra” biofunctions compared to the previous hierarchy were presented in this figure

We then compared DMGs in *A. squarrosum* with other plants, including a dicot model plant *A. thaliana* [21], *Z. mays* [34], and a perennial monocot, *P. virgatum* [13] (Table 1). Efforts were made to figure out the key element shaping the strong drought memory in *A. squarrosum*. Ortholog groups (OGs) seeking were performed between the three previously studied species and two ecotypes of *A. squarrosum* (Fig. 2a, Additional File 8). We focused on the following four non-overlapping gene hierarchies: 257 DMG OGs shared among the five tested species, which might underpin the molecular mechanism of dehydration stress memory in most higher plants; 406 AS (short for *A. squarrosum*) DMG OGs shared between AEX and WW, namely unique in *A. squarrosum*, which possibly helps modeling *A. squarrosum*’s peculiar drought memory; 655 and 339 OGs particularly in AEX DMG and WW DMG, respectively (Fig. 2a), which could be the reason why there were discrepancies between the two ecotypes. KEGG enrichment analyses of non-memory genes and late-responsive genes in Table 1 were also conducted (Additional File 9).

Gene Ontology (GO) and KEGG enrichment analyses of the four distinct hierarchies mentioned above were then carried out to clarify their potential function (Fig. 2b and c). Shared DMGs were enriched in the following GO terms: apoplast (GO:0048046), cell wall (GO:0009505), heme binding (GO:0020037), and responses to stimulus like chitin (GO:0010200), karrikin (GO:0080167), and heat (GO:0009408). GO functions enriched in AS DMG were mainly responses to environmental cues, for instance, heat (GO:0009408), high light intensity (GO:0009644), hydrogen peroxide (GO:0042542), and water deprivation (GO:0006457); from which we might draw the inference that more stress-related genes have been recruited by *A. squarrosum* than other plant species to reinforce drought memory. As to DMGs unique in AEX, multiple functions were involved in stress toleration, including lipid droplet, signaling, hormone activity, etc. In contrast, the majority of WW DMG was concentrated on photosynthesis-related functions (Fig. 2b).

KEGG enrichment analysis showed that plants shared DMGs were distributed in phenylpropanoid biosynthesis (ko00940), MAPK signaling pathway (ko04016), saccharide metabolism and interconversions (ko00500, ko00040), plant-pathogen interaction (ko04626), etc., while AS DMGs mainly involved in protein processing in endoplasmic reticulum (ko04141), galactose metabolism (ko00052), plant-pathogen interaction (ko04626), and glutathione metabolism (ko00480). For AEX DMGs, the metabolism of inositol phosphate (ko00562), alanine, aspartate, glutamate (ko00250), and glutathione (ko00480) metabolism were mainly enriched; while in WW DMGs, the three most abundant terms were glutathione metabolism (ko00480), photosynthesis (ko00195, ko00196), and glycerophospholipid metabolism (ko00564, Fig. 2c).

**Fig. 3:**
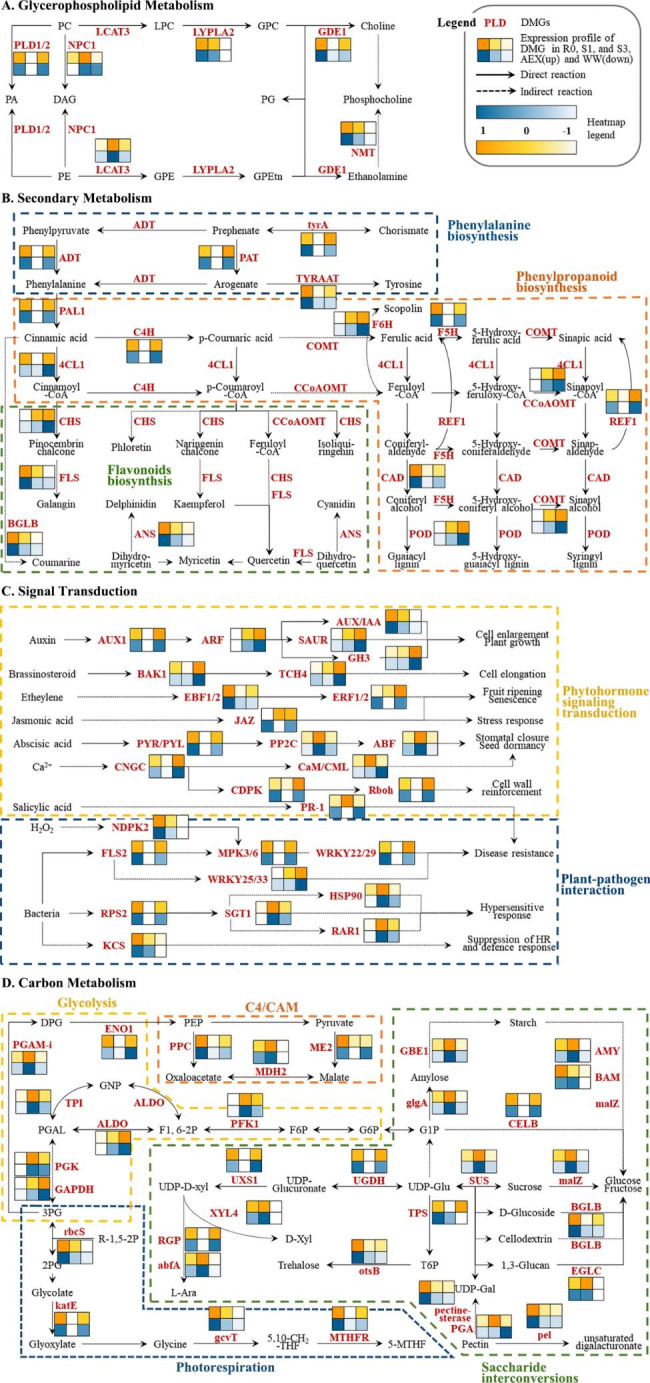
Four main metabolism networks in which *A. squarrosum* DMG enriched. **(a)**, glycerophospholipid metabolism; **(b)**, secondary metabolism; **(c)**, signal transduction; **(d)**, carbon metabolism. Heatmap showed the expression profiles of the corresponding DMGs in R0, S1, and S3, where the upper yellow hues and the under blue hues represented AEX and WW, respectively. Modules were framed in dashed boxes of different colors

Next, four dominantly enriched metabolism networks, glycerophospholipid metabolism, secondary metabolism, carbon metabolism, and signal transduction, in which all the *A. squarrosum* DMGs involved were generated (Fig. 3). In glycerophospholipid metabolism pathway, six DMGs encoding key enzymes showed different expression, whereby *phospholipase D1/2* (*PLD1/2*, EC:3.1.4.4) and *glycerophosphodiester phosphodiesterase* (*GDE1*, EC:3.1.4.46) could be categorized into [−/+], *non-specific phospholipase C1* (*NPC1*, EC:3.1.4.3) and *phospholipase A1* (*LCAT3*, EC:3.1.1.32) were in the group [+/−], while *lysophospholipase II* (*LYPLA2*, EC:3.1.1.5) and *phosphoethanolamine N-methyltransferase* (*NMT*, EC:2.1.1.103) in [−/−], resulting in discrepancy in accumulation of plant cell membrane ingredients, i.e., phosphatidylcholine (PC), phosphatidylethanolamine (PE) and choline, and signaling molecules, such as 1,2-diacylglycerol (DAG) and 1,2-diacyl-sn-glycerol 3-phosphate (PA), under different water condition (Fig. 3a).

DMGs in *A. squarrosum* were found to take pervasive parts in secondary metabolism (Fig. 3b). In this section, most DMGs were sorted as [−/+], including *arogenate/prephenate dehydratase* (*ADT*, EC:4.2.1.91/4.2.1.51), *chorismate mutase* (*tyrA*, EC:5.4.99.5), *aspartate aminotransferase* (*PAT*, EC:2.6.1.1), *arogenate dehydrogenase* (*TYRAAT*, EC:1.3.1.78) in phenylalanine biosynthesis, and *phenylalanine ammonia-lyase* (*PAL*, EC:4.3.1.24), *4-coumarate–CoA ligase* (*4CL*, EC:6.2.1.12), *trans-cinnamate 4-monooxygenase* (*C4H*, EC:1.14.14.91), *ferulate-5-hydroxylase* (*F5H*, EC:1.14.-.-), *cinnamyl-alcohol dehydrogenase* (*CAD*, EC:1.2.1.68), *coniferyl-aldehyde dehydrogenase* (*REF1*, EC:1.2.1.68) in phenylpropanoid biosynthesis pathway. However, expression profiles of DMGs in flavonoids biosynthesis, i.e., *flavonol synthase* (*FLS*, EC:1.14.20.6), *beta-glucosidase* (*BGLB*, EC:3.2.1.21), and *anthocyanidin synthase* (*ANS*, EC:1.14.20.4), went through a continuous decline along treatments (category [−/−]). In addition, *caffeoyl-CoA O-methyltransferase* (*CCoAOMT*, EC:2.1.1.104) and *peroxidase* (*POD*, EC:1.11.1.7), the two key enzymes in lignin biogenesis, as well as *feruloyl-CoA 6-hydroxylase* (*F6H*, EC:1.14.11.61), which catalyzes the synthesis of scopolin, were identified to have amplified effects in S1 and subsequent S3 (category [+/+]).

DMGs in *A. squarrosum* acted as receptors and effectors of multiple phytohormones including auxin, abscisic acid (ABA), brassinosteroids (BRs), ethylene (ETH), jasmonic acid (JA), and salicylic acid (SA, Fig. 3c). Auxin and BRs transduction were activated by repeated drought treatment, for expression profiles of most DMGs involved in these two pathways were [+/+], such as *auxin response factor* (*ARF*), *SAUR family protein* (*SAUR*), *auxin-responsive GH3 gene family* (*GH3*), and *xyloglucan: xyloglucosyl transferase* (*TCH4*, EC:2.4.1.207). Conversely, DMGs in other signal transduction, for instance, ETH, JA, ABA, Ca^2+^, and SA, showed reversed expression levels between S1 and S3, either [+/−] or [−/+]. This pattern was also found in the majority of DMGs related to plant-pathogen interaction. Among them, three disease resistance-related genes, *LRR receptor-like serine/threonine-protein kinase* (*FLS2*, EC:2.7.11.1), and members of two transcription factors, *MPK3/6* and *WRKY22/29* displayed [−/+] mode, while three genes specifically induce hypersensitive reaction (HR, a part of the defense response against pathogen attack), that were *suppressor of G2 allele of SKP1* (*SGT1*), *heat shock protein* 90 kDa (*HSP90)*, and *disease resistance protein* (*RAR1*), were sorted in category [+/−]. *3-ketoacyl-CoA synthase* (*KCS*, EC:2.3.1.199), the coding gene of a repressor of HR and defense response, was one of the exceptions, which presented consecutive decreasing along S1 and S3 (category [−/−], Fig. 3c). The results illustrated that recurring dehydration treatment arose crosstalk between resistance to biotic and abiotic stress in *A. squarrosum*.

A rather complex network of carbon metabolism was associated with *A. squarrosum* DMGs, harboring four sections: glycolysis (EMP), C4-dicarboxylic acid cycle/Crassulacean acid metabolism (C4/CAM), photorespiration, and saccharide interconversions (Fig. 3d). The recurring drought treatment activated EMP, whereas weakened photorespiration process, which presented as most enzymes in the two mentioned pathways were significantly up-regulated and down-regulated when compared to R0, respectively. In C4/CAM pathway, the two enzymes, NADP-dependent malic enzyme (ME1, EC:1.1.1.40) and malate dehydrogenase (MDH2, EC:1.1.1.37), catalyze the reaction from pyruvate to malate, and the conversion between oxaloacetate and malate, respectively, were found in subtle balance in S1 and S3. An interconversions network with UDP-glucose as the center was generated based on *A. squarrosum* DMGs, which mainly referred to the transformation of UDP-glucose to soluble monose or disaccharide (i.e., D-glucose, D-fructose, sucrose, trehalose, and pentose), as well as to the regulation of pectin contents in the plant. Most enzymes catalyzing transformation to sucrose, glucose, fructose, and xylose had the maximum expression level at the first drought, while those responsible for arabinose synthesis were down-regulated in S1 but up-regulated in S3. Pectin degradation and trehalose biosynthesis were both decreased, resulting in pectin accumulation and less trehalose generated, under multiple drought treatments.

Moreover, the expression profiles of some photosystem subunits-coding DMGs have changed under repeated drought (Additional File 10a); changes in subunits of electron transport chain in oxidative phosphorylation were also found (Additional File 10b), indicating that *A. squarrosum* drought memory morphogenesis was a widespread process existed in the central metabolic system, which was consistent with previous research [18].

### Protein-protein interaction (PPI) network in ***A. squarrosum*** DMGs

**Fig. 4:**
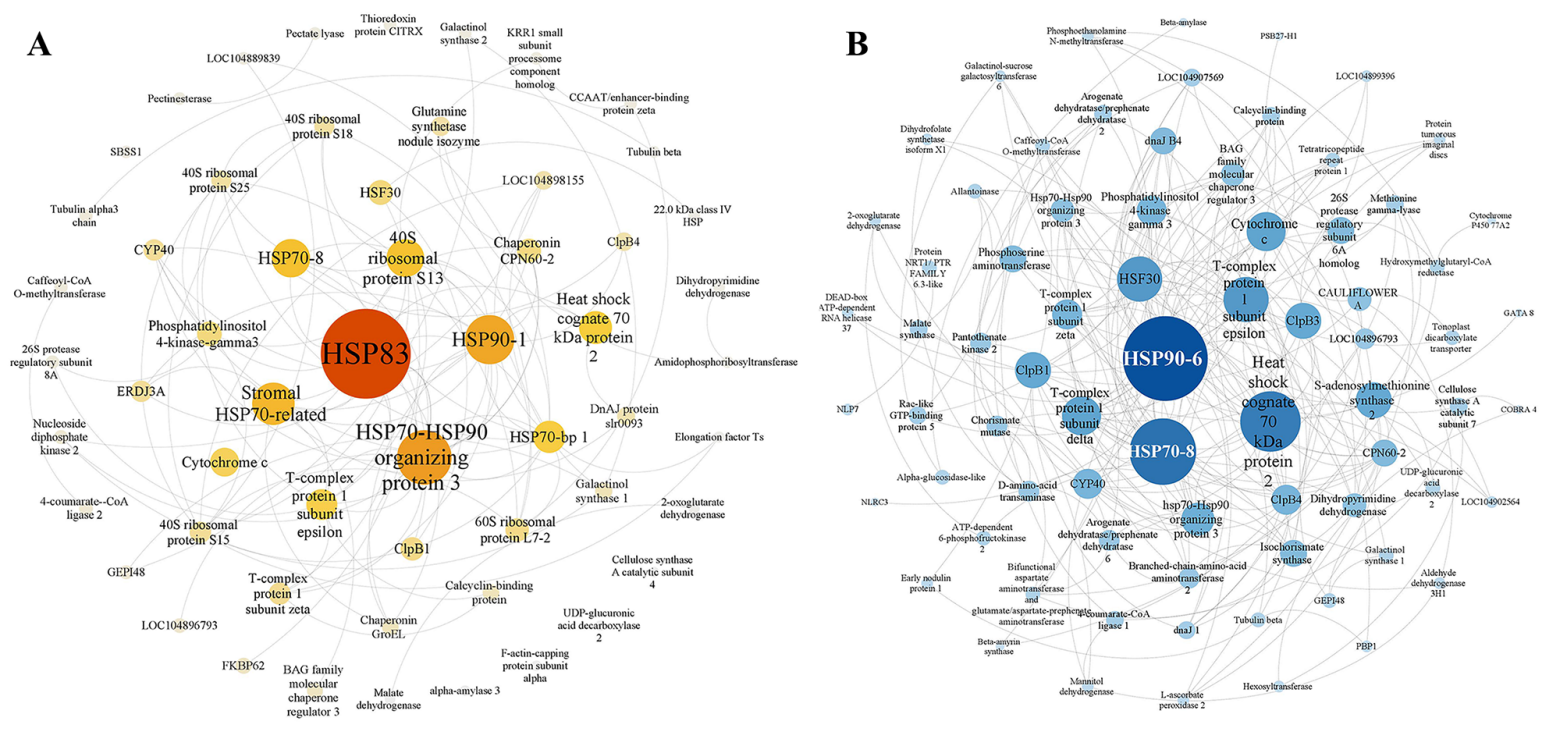
PPI network of DMG in AEX **(a)** and WW **(b)**. PPI networks in AEX and WW were presented in yellow and blue hues, respectively. Hub genes in each network were rated from large (central) to small (edge)

PPI networks based on DMGs of AEX and WW were constructed separately to elucidate interactions of protein-coding genes responsible for drought memory in *A. squarrosum* (Fig. 4). In AEX, HSP83, HSP70-HSP90 organizing protein 3, HSP90-1, stromal HSP70-related protein, and HSP70-8 were top five proteins with highest connectivity score (Fig. 4a), making them hub genes in protein interaction network; while in WW, this list was constituted of HSP90-6, HSP70-8, heat shock cognate 70 kDa protein 2, T-complex protein 1 subunit epsilon, and HSF30 (Fig. 4b, rankings were generated by cytohubba), which indicated that HSP-related proteins were of highly importance in drought memory for both ecotypes.

### Possible molecular switches in ***A. squarrosum*** drought memory

Due to its excellent water use efficiency in deserts, we are expecting to see clues of a refined drought memory regulatory network in *A. squarrosum*. A large number of genes may be driven or deactivated by some kind of signal simultaneously when repeating drought occurs. In this regard, TFs that regulate the expression levels of hundreds of target genes (in the case of global TFs, thousands of) are the optimal candidate regulators. A total of 71 drought memory transcription factors (TFs) or transcription regulators (TRs) were identified and classified according to the four DMG hierarchies, among these, were 43 in shared DMGs, nine in AS DMGs, whereas extra 12 and 13 in AEX and WW DMGs, respectively (Additional File 11). In total, we found 14 members in AP2/ERF family, twelve in MYB, nine in WRKY, three in C3H, GARP-G2, and MADS-MIKC, respectively. In addition, some TRs were discovered only in AS and ecotype unique DMGs, such as *AsIAA31*, *AsMBF1c*, and one member in SET family, indicating that *A. squarrosum* has recruited extra transcription regulators to enhance its drought memory.

**Fig. 5:**
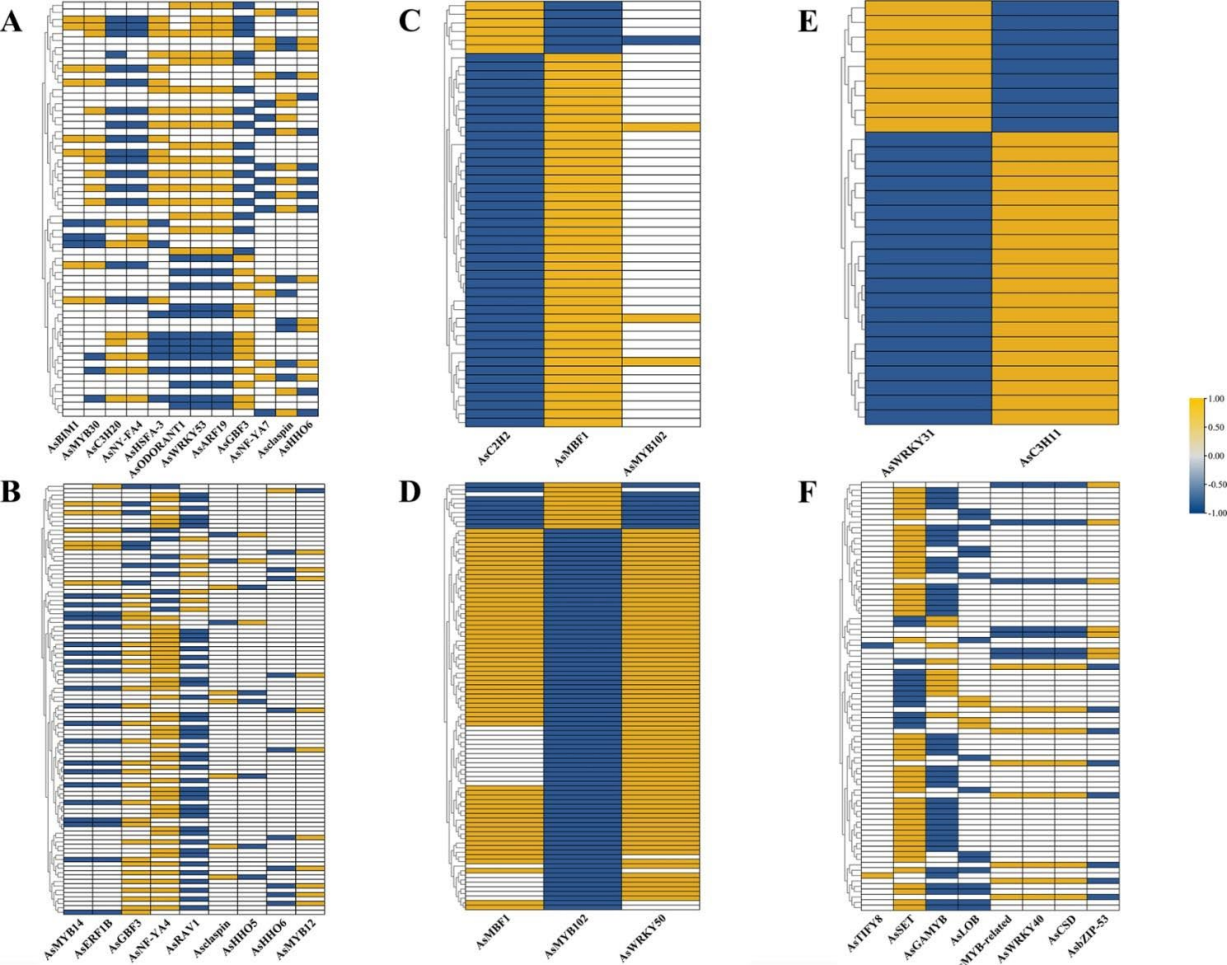
Co-expression analysis between drought memory TFs and the rest DMGs within the same hierarchy across the two ecotypes. **(a)**, shared DMG expressed in AEX; **(b)**, shared DMG expressed in WW; **(c)**, AS DMG expressed in AEX; **(d)**, AS DMG expressed in WW; **(e)**, ecotype-unique DMG expressed in AEX; **(f)**, ecotype-unique DMG expressed in WW. This figure only presented strong co-expression with Pearson correlation coefficient > 0.9 and p-value < 0.05, where positive correlation and negative correlation were depicted as yellow and blue, respectively

To elucidate the regulatory model of TFs, within each of the four hierarchies across ecotypes, gene co-expression analysis between drought memory TF/TRs and the rest DMGs was conducted based on Pearson correlation coefficient. Interestingly, in every hierarchy each ecotype, a quite neat positive-negative correlation was found between TFs and at least a couple of DMG sets, as if these DMG sets were controlled to switch between ‘on’ and ‘off’ by a same pair of TFs (Fig. 5). For instance, in shared DMG expressed in AEX (Fig. 5a), 30 DMGs were subjected to a positive-negative regulatory module consisted of *AsGBF3-AsODORANT1-AsWRKY53-AsARF19*, whereas in shared DMG in WW, a major module consisted of *AsNF-YA4-AsRAV1* controlled a set of 48 DMGs, another three modules consisted of *AsMYB14-AsERF1B-AsGBF3*, *Asclaspin-AsHHO5*, and *AsHHO6-AsMYB12*, respectively, were also found (Fig. 5b). In AS DMG in AEX, three TFs, *AsC2H2*, *AsMBF1*, and *AsMYB102*, built up a regulatory module on 49 DMGs (Fig. 5c), while in the same hierarchy in WW, *AsMBF1*, *AsMYB102* and *AsWRKY5* were in responsibility for the expression of a set of 75DMGs (Fig. 5d). An *AsWRKY31-AsC3H11* module found in AEX DMG showed exactly opposite regulation on a same set of 29 genes (Fig. 5e); two positive-negative controlling modules comprised of *AsSET-AsGAMYB-AsLOB40* and *AsRADIALIS3-AsWRKY40-AsCSD-AsbZIP53*, respectively, were found in WW unique DMG, in which *AsSET* and *AsbZIP53* might be considered as the “key switch”, for they required the other two or three TFs to balance their possible biological function (Fig. 5f). In total, 17 and 20 TFs played the role of ‘on-off’ controlling in AEX and WW, respectively.

On the other hand, DMG sets subjected to the TF switches were 137 and 280 in AEX and WW, respectively, in their corresponding hierarchies. These DMGs embraced a bunch of uncharacterized genes, and functions of which were fragmented (Additional File 12). Nevertheless, large number of coding genes of proteins or enzymes, which not only play important roles in stress defense, but essential for plant vital life activities, were also found to be subjected to TF switches, that among these, were heat shock proteins (HSPs), chaperonin (e.g., Clp, late embryogenesis abundant protein (LEA), etc.), cytochrome P450, mitogen-activated protein kinase (MAPK), LRR receptor-like serine/threonine-protein kinase, glutathione S-transferase, and so forth (Additional File 13).

### Q-PCR validation of the transcriptome data

**Fig. 6:**
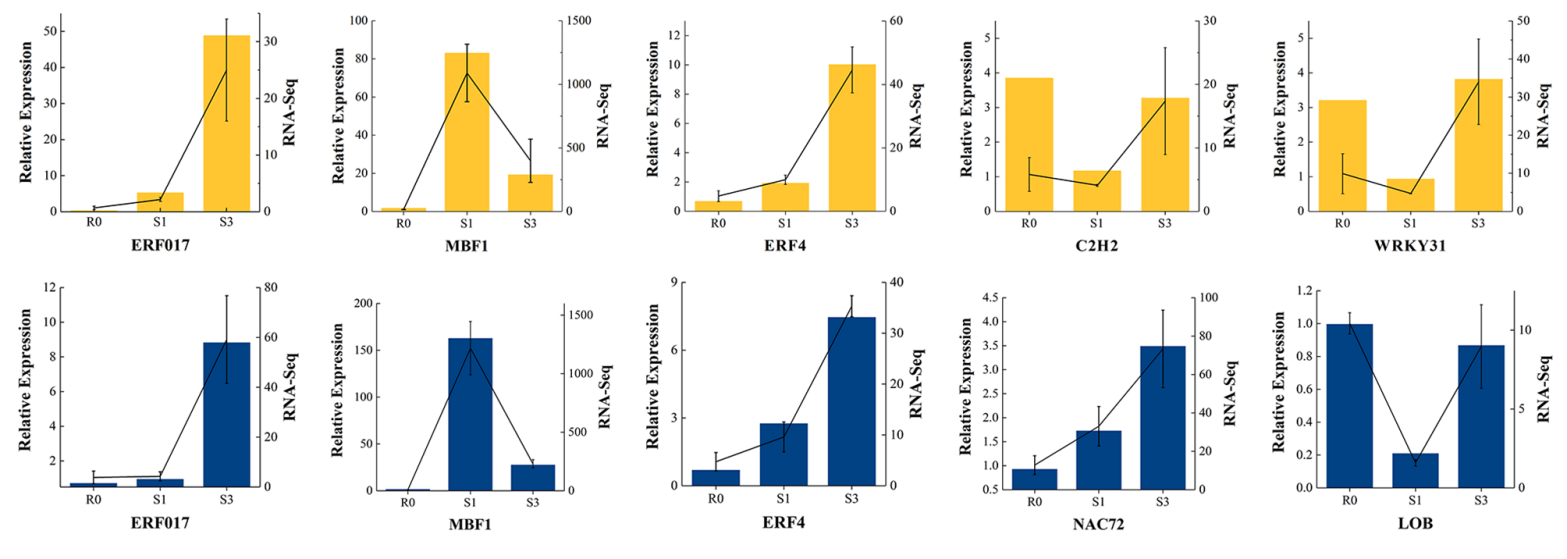
Q-PCR validation of the transcriptome data. The histogram and the line graph with error bar indicates FPKM generated by RNA-Seq and the relative expression calculated by 2^−ΔΔCt^ method, while yellow and blue graphs represent gene examined in AEX and WW, respectively

The authenticity of the transcriptome data generated in the present research was validated by real-time fluorescence quantitative PCR. The relatively expression profiles of 10 TFs were of highly correlation with NGS data, confirming the reliability and repeatability of the transcriptome data (Fig. 6).

## Discussion

### Commonalities of general higher plants and characteristics of ***A. squarrosum***in drought memory

In this research, we compared DMGs in *A. squarrosum* and previous studied plants, including dicots model plant *A. thaliana* and two monocots, *P. virgatum* and *Z. mays*. The DMG intersection from these species may at least partially underpin the common nature of drought memory in higher plants. Phenylpropanoid biosynthesis, cyanoamino acid metabolism, and glutathione metabolism were enriched in all the four DMG hierarchies, indicating their irreplaceable roles in plants drought memory (Fig. 4). A great quantity of secondary metabolites, such as phenolic compounds, flavonoids, anthocyanins and lignin can be induced by drought stress, where phenylpropanoid biosynthesis plays a central role in these pathways [35]. Limited studies on cyanoamino acid supported that cyanoalanine, the product of cyanide detoxification, was identified as potential biological markers of drought response [36], and that cyanoamino acid metabolism pathway could be activated by water deficit, exogenous H_2_S under drought [37], pathogen [38], heavy metal [39], and was also related to seed coat pigmentation in *Brassica napus* [40]. *“*Glutathione metabolism” mainly referred to glutathione-S-transferase (GST) in the present research, for there were 19 copies of GST family members in DMG gene set (Additional File 5). GSTs catalyzes the conjugation of electrophilic compounds to an essential antioxidant, glutathione (GSH), and regulates the GSH pool in vacuoles or apoplast [41]. Increased GST levels maintain cell redox homeostasis and protect organisms against oxidative stress. Experimental evidences suggested that GST increased plants oxidative stress tolerance: *atgstu17* mutated *A. thaliana* were more tolerant to drought and salt stresses compared with wild-type [42], indicating a role of AtGSTU17 in adaptive responses to drought and salt stresses by functioning as a negative component of stress-mediated signal transduction pathways. In *O. sativa*, *OsMADS25* increases the ROS-scavenging capacity by activating the expression of *OsGST4* directly [43]. GST-coding genes were highly expressed only in drought-tolerant genotype of *Hordeum vulgare*, and was thought to play important roles in detoxification, thus increased drought tolerance [44].

On the other hand, progressive relationship in the four DMG hierarchies showed in Venn diagram (Fig. 2a), and that barely any overlapping function was found among the four hierarchies (Fig. 2b and c) supporting the fact that *A. squarrosum* recruited more distinct genes compared to other species in drought memory. On the basis of prevalent defense reaction of shared DMGs, additional GO terms related to environmental abiotic stimuli were largely abundant in AS DMGs, which endowed *A. squarrosum* with a more powerful defense when facing adverse condition. It might be due to its in situ adaptation to the complex environment factors in desert integrating intense sunlight, high temperature and water deficiency. Furthermore, discrepancy of GO terms enriched in ecotype-unique DMG depicted that different acts were taken by the two contrasting ecotypes under the same condition. The enrichment of AEX DMG was rather fragmented, while WW DMG was mainly enriched in functions relative to photosynthesis (Fig. 2b). Photosynthesis is one of the most vulnerable biological processes under dehydration [45]. Drought stress causes an increase in ABA synthesis, leading to stomatal closure and an alteration in photophosphorylation, including change in the composition of thylakoid membrane protein, chlorophyll content, the amount of ATP, thus leading to a decreased regeneration of RuBP, which not only negatively affects the light reactions, but also the assimilation efficiency of the dark reactions, thereby reducing the contents of the photosynthetic products [46, 47]. Whether WW ecotype take steps to cope with the upcoming dehydration after multi-drought stress training, such as changing the structure of thylakoid membrane and the activity of photosystem complex, acquires further research. What is known, however, is that expression of some photosystem subunits-coding DMGs have changed under repeated drought (Additional File 10a). In brief, in accordance with physiological observation (Fig. 1), a relatively stronger stress memory mechanism was triggered in WW and endowed the plants with the ability to react more persistently to the same adverse condition, which might be a sign of adaptation to, or “training result” of, long and rigorous recurring water stress in its original arid habitat.

### Fine tune in drought memory-related metabolism pathways in ***A. squarrosum.***

Trade-off was observed in-between some of the drought memory pathways during the repeating drought treatment, indicating *A. squarrosum* has evolved fine adjustment in drought memory on the transcriptional level (Fig. 3). For instance, genes related to flavonoids biosynthesis went through continuous down-regulation, while the key enzymes in lignin biosynthesis were consecutively up-regulated after recurring drought treatment (Fig. 3b), which means *A. squarrosum* preferred lignin as the defense compound. Flavonoids and lignin are the two main branches of phenylpropanoid biosynthesis pathway, the metabolic flux redirection (MFR) between lignin and flavonoids biosynthesis is one of the key issues in plant secondary metabolism research [48]. Lignin is a highly branched polymer of phenylpropanoid compound and one of the main components of plant cell wall. It has been reported that expression of key enzymes in lignin biosynthesis, such as CCoAOMT, PAL, 4CL, CAD, POD, etc., can be induced by drought stress [49–54]. Flavonoids, on the other hand, are parts of non-enzymatic antioxidants system, and suggested to be defense metabolites against environmental stress [55]. Generally, water shortage accelerates flavonoids accumulation [56–60]. Integrated analyses of multi-omics provided evidence of reduced lignin synthases but increased flavonoids synthases under water deficit in Qingke (highland barley) [61], *Camellia sinensis* [62], and *O. sativa* [63]. In contrast, in our previous studies, the expression of lignin biosynthesis genes was up-regulated in a lowland ecotype of *A. squarrosum* after transplanting to a middle-altitude common garden, which was speculated to be the consequence of stress response [64]. One of the possible explanations to the question of why there is transcriptional trade-off between lignin and flavonoids, the two compounds of great value in defense reaction, is that *A. squarrosum* might have found the optimal solution to take full advantage of limited resources in desert under unpredicted repeated drought stress, for lignin provide mechanical support and a more prevalent defense against multiple biotic/abiotic stresses than flavonoids.

Saccharide interconversion that facilitates adaptive changes in carbon allocation plays a profound physiological role of protection against environmental stresses [65]. Refined regulation was found in carbon metabolic pathway from UDP-glucose to multiple conversion outlets, including pectin, hemicellulose (mainly xylose and arabinose), trehalose, and three principal sugars (sucrose, glucose, and fructose; Fig. 3d). Primary cell walls, of which hemicellulose and pectin are the major components, generate turgor pressure, accommodate cell expansion, mediate cell adhesion, and thus play an important role in plant defense and responses to environmental stresses [66]. A few researches showed that plants may adjust the ratio of xylose and arabinose during stresses, and that composition alters may lead to changes in primary cell wall structure: increased xylose and decreased arabinose were monitored separately in *Quercus suber* bark after water shortage [67]; in *A. thaliana*, mutants with mutations for reduced xylose and galactose was the least drought tolerant, while the arabinose-altered mutants were the least affected by water loss [68]; 30℃ of heat stress lowered the content of xylose, while under 70℃ treatment, xylose and arabinose were elevated and reduced separately in *C. arabica* leaves [69]. It is widely accepted that sucrose and glucose accumulation increase cell osmotic potential, maintain cell turgor, and enhance water uptake, and are critical components in maintenance of cell wall integrity and the regulation of induced responses. Under recurring drought stress, the [+/−] mode of sucrose/glucose synthases, the [−/+] mode of xylose/arabinose synthases, as well as the pectin accumulation resulted in dynamic changes of these saccharide contents in different treatment stages, and concerted the response to water deficit in *A. squarrosum*. On the other hand, as a non-reducing rare disaccharide with abilities of energy source or osmolyte, trehalose is regarded as a global protectant against abiotic stresses [70, 71]. It is reported that the primary effect of trehalose is not as a compatible solute but both a signal and a negative feedback regulator of sucrose levels in plants, correlating with higher soluble carbohydrate levels and an elevated capacity for photosynthesis under both stress and nonstress conditions [72]. Trehalose biosynthesis is a two-step enzymatic process, where trehalose-6-phosphate (T6P) is synthesized from UDP-glucose by trehalose-6-phosphate synthase (TPS) and subsequently converted to trehalose by trehalose-6-phosphate phosphatase (TPP) [73]. T6P is a central sugar signal in plants, regulates sucrose use and allocation, and thus regulates plant’s tolerance to drought [70]. It is suggested that the proper regulation of T6P accumulation by TPS and TPP is key to enhance the adaptability to drought in *Glycine max* [74]. In *A. squarrosum*, the expression of *TPS* in AEX ecotype went down at S1 then up at S3, while that of WW ecotype went continuously down; in contrast, the expression of TPP (otsB in this research, a bacterial-originated isozyme of TPS) coding-gene was down-regulated after recurring drought in both two ecotypes, leading to the refined adjustment of T6P accumulation, thus promote its regulation role. Additionally, the Sucrose-T6P nexus in plants could be a partial explanation of the expression fluctuation of *TPS* and *TPP* in *A. squarrosum* after recurring drought, that a negative feedback loop comprised of sucrose and T6P, whereby any increase or decrease in T6P leads to an opposite change in sucrose levels [72]. Fully understanding on how drought memory-related metabolism network functions necessitates combination of multiple omics tools in *A. squarrosum*, which will be our future research direction. In brief, these findings further illustrate the fine tune of multiple metabolic pathways in vivo when plants cope with recurring water deficit.

### Possible “molecular switch” in ***A. squarrosum*** drought memory

A possible molecular switch mechanism constituted of pairwise TF/TR was observed in the present research. Quite neat positive-negative pair-wise co-expression generated by Pearson correlation coefficient with p < 0.05 in Fig. 5 illustrating the potential function of TFs (or TRs) pairs as molecular switches. Although Liu et al. suggested that the TF memory behavior is not the general mechanism imparting the memory transcriptional patterns to all regulated genes in *A. thaliana* (strongly supported by the drought-memory mode of MYC2 and the non-drought memory mode of its direct binding target, *RD22*) [75], this phenomenon was even observed in every hierarchy across the two *A. squarrosum* ecotypes, which was unlikely by mere odds.

To our knowledge, no evidence was found that multiple TF/TR pairs could function as molecular switches. The general form of molecular switches in plants may contain the following four: (1) feedback regulation loop [76–78]; (2) modulating downstream reaction via transform between active/inactive state [79, 80]; (3) mediating different effects by substrates preference altering [81]; (4) single genes that control vital life activities or mediate a series of effects. [82–86]. Here we offered a new perspective, that a pair of TFs may function as “on-off” molecular switch in at least partial *A. squarrosum* drought memory behavior, e.g., *AsC2H2*-*AsMBF1* in Fig. 5c, *AsC2H2*-*AsMBF1-AsWRKY50* in Fig. 5d, and *AsWRKY31-AsC3H11* in Fig. 5e, controlling the expression level of a same gene sets to alter between high (on) and low (off), and thus rendering the ability of plant to convert between “remember” and “forget”. Based on this mechanism, the plant could maintain the homeostatic balance between plants development and energy-consuming defense reaction.

**Fig. 7:**
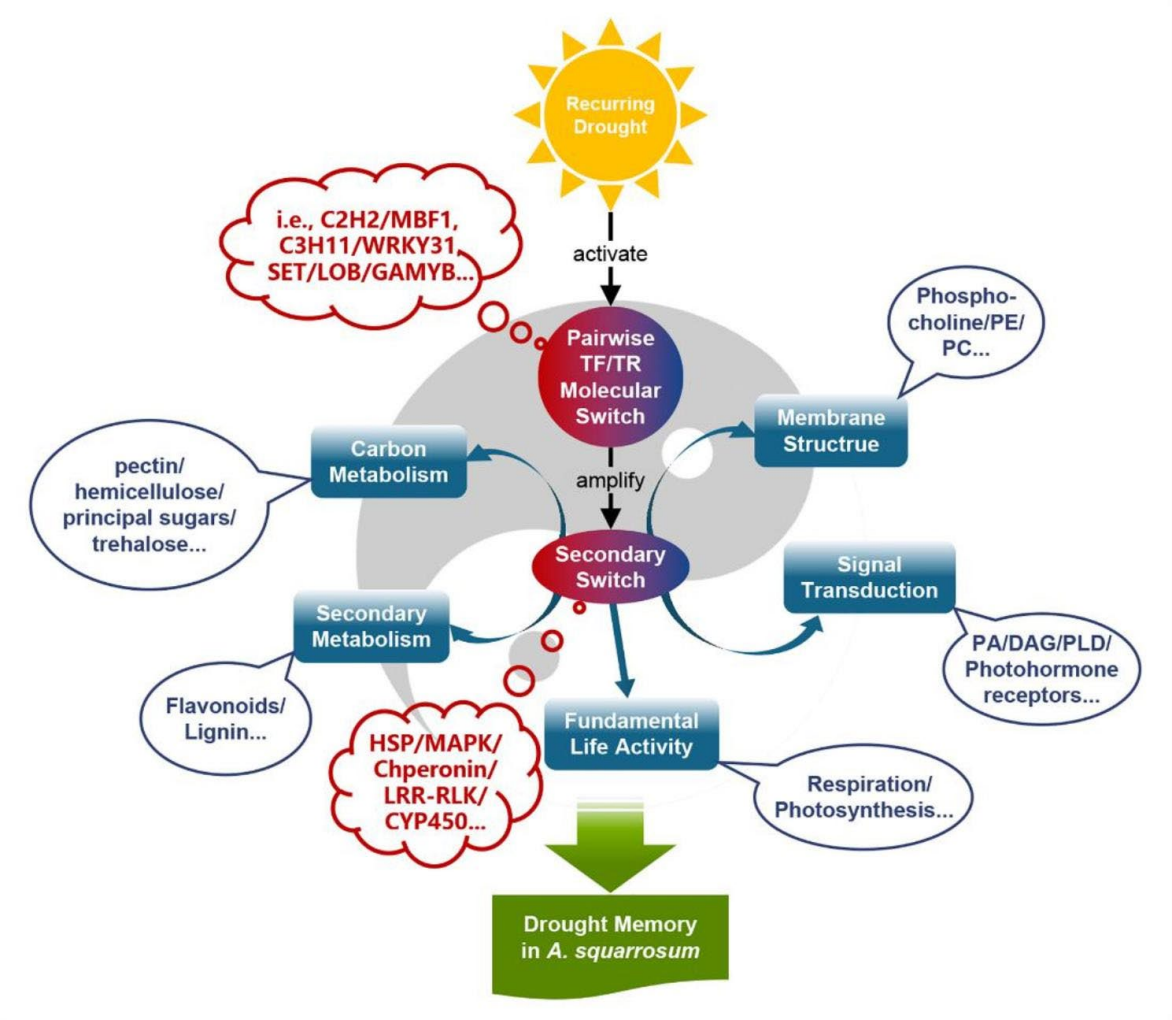
Possible regulatory module of drought memory in *A. squarrosum*

Additionally, the fact that HSPs took the central part in PPI network of DMGs in both two *A. squarrosum* ecotypes (Fig. 4) might leading to the assumption that these TF switch-controlled DMGs, especially HSPs, act as amplifiers, or secondary switches, in modulating downstream drought memory mechanism by transducing as well as augmenting the TF switch signal. Hence, a possible module of *A. squarrosum* drought memory is taking shape: recurring drought activates TF/TR molecular switches, and this signal is amplified by the secondary switches, thereby modulating fine tunes of downstream metabolic pathways including fundamental life activities, signal transduction, membrane structure, carbon metabolism and secondary metabolism, which endows the plant with faster and/or stronger reaction to the unpredictable repeated water stress (Fig. 7). To be mentioned, not all the DMGs subjected to TF switches are their direct targets. Plants mobilize multiple biological processes by regulating enzyme-coding genes activities on the transcriptional level, which are mainly regulated by TFs. However, it is rather difficult to infer direct regulation between TFs and their targets by using their expressional profiles independently [87]. One of the examples is Multiprotein Bridging Factor 1 (AsMBF1), the key TF switch in AS DMG (Fig. 5c and d). MBF1 is a non-DNA binding transcription co-factor, whose molecular function is to form a bridge between TFs and the basal machinery of transcription, TATA box binding protein (TBP). The MBF1-TBP-TF ternary complex is involved in multiple developmental processes and in stress responses [88]. In this case, there is no other way to figure out the direct target of AsMBF1 but to rely on the co-expression profiles. Although the artificial classification of DMGs may lead to the TF switches to be overrated, missing, or underestimated to some extent, and the drought memory module we proposed here remains many gaps to be filled, we believe this conceptional module can be at least a partially explanation of drought memory genesis in *A. squarrosum*.

## Conclusion

In this study, the outperformance of drought memory in arid land-ecotype WW than semi-arid land-ecotype AEX of a psammophyte *A. squarrosum* was proved by physiological observations after recurring drought treatment. Compared to the previously researched species, *A. squarrosum* motivated more and distinct DMGs in drought memory mechanism, whose function was mainly related to defense responses to heat, high light intensity, hydrogen peroxide, and water deprivation, while DMG functions in AEX and WW showed discrepancies, indicating that long-term adaptation to water heterogeneity may lead to different drought memory strategies. We proposed a possible module of drought memory generated in *A. squarrosum*, whereby pairs of TF/TRs that control the expression level of same gene sets may function as positive-negative molecular switches in drought memory, and regulate fine tune of plant’s multiple vital activities and complex metabolism networks, including signal transduction, photosynthesis, oxidative phosphorylation, carbon metabolism, and secondary metabolism. Demonstration of the spatial-temporal regulation of TF switch needs further research. In this regard, the availability of promotor region details in our upcoming *A. squarrosum* genome will assist clarifying the interaction between TFs and their candidate target genes; in vitro validation, like yeast one-hybrid or CO-IP technique, is also required.

## Materials and methods

### Plant growth, treatments, and sample collection

Two contrasting ecotypes, AEX (semi-arid land ecotype) and WW (arid land ecotype), of *A. squarrosum* (L.) Moq. plants were used in the present research. Seeds of AEX ecotype were originally collected from a semi-arid site Aerxiang (Northeast China, Horqin Sand Land, 42°52′4.80″N, 122°25′40.14″E), while those of WW ecotype were collected from an arid region in Wuwei (Northwest China, Tengger Desert, 37°54′10.98″N, 102°54′4.2″E), where annual mean precipitation is 485 mm and 166 mm, with annual mean temperatures of 6.38℃ and 7.9℃, respectively. The voucher specimens for wild seeds of AEX and WW are reserved in the *A. squarrosum* seed bank at Northwest Institute of Eco-Environment and Resources, Chinese Academy of Sciences, with voucher ID AEX2019-25-2 and WW2019-13-3, respectively. Seeds were grown in pots (sizes: upper diameter, 21 cm; height, 16 cm; bottom diameter, 11 cm) filled with soil (loess) and sand (1:1) with photoperiod 16/8 h, temperature 30℃/22℃ in the greenhouse at Northwest Institute of Eco-Environment and Resources (NIEER, CAS) for four weeks, to simulate the natural growth condition in the wild. Twenty pots of one-month-old seedlings with consistent growth were separated evenly into the control group and experiment group, across ecotypes. Water was continuously provided to the control group throughout the trial so that soil moisture was close to the maximum field capacity, while the experiment group was subjected to three rounds of recurring drought stresses/re-watering cycle, which was determined as a four-day of water suspended followed by a five-day fully re-hydration recovery in each cycle. For control group and rehydration treatment, the pots were sunk in a shallow plate full of water at around 10 am every other day (e.g., Day 1, 3, 5 in each rehydration period) so that the loess-sand mixture can uptake water to the maximum field capacity. Mature leaves pooled from five individual plants for each ecotype were collected in the control group as R0, and by the end of each drought stress and recovery processes (S1/R1, S2/R2, and S3) in the experiment group (Fig. 1a). All leaves were sampled right before the next treatment cycle. For example, sample S1 was detached at 9 am on R1 Day1, just right before the 1st rehydration treatment, while sample R1 was detached at the same time on S2 Day1.

### Physiological and morphological measurements

Soil moisture content (SMC), leaf relative water content (Leaf RWC), and water loss of isolated leaves were employed to evaluate drought resistance and robustness of stress memory between the two ecotypes of *A. squarrosum* in the recurring dehydration/re-hydration cycle. Soil moisture content of each stage was monitored right before the next treatment (the same time as leaves sampling) throughout the experiment using a soil moisture sensor (Shun Koda TR-6, China). Fresh weight (FW), turgid weight (TW), wilted weight (WW), and dry weight (DW) from each leaf sample were weighed immediately after being isolated from the plants, water-saturated at 4℃ in darkness for 24 h, spread on a laboratory bench to wilt at room temperature (20℃) for 6 h, and dried at 70℃ for 72 h, respectively. Leaf RWC was calculated according to the formula leaf RWC (%) = (FW-DW)/(TW-DW)×100. Leaf water loss was determined using the formula leaf water loss (g/g) = (FW-WW)/DW [89].

Physiological data were statistically analyzed using the analysis of variance (ANOVA) procedure and t-test at 0.05 level of IBM SPSS Statistic 25 (SPSS Inc., Armonk, NY, USA). Data with replicates were analyzed and presented as the mean of three or more replicates ± SD. All figures were produced using the software Origin 2017 (Origin Lab Corporation).

### RNA extraction and RNA-seq library construction

RNA sequencing was performed to understand the molecular mechanism of drought memory in *A. squarrosum*. Drought memory genes were defined as drought-responsive genes with significant expression changes among recurring dehydration treatments, thus, mature leaves from five individuals of each control, the first drought treatment, and the third drought treatment (namely R0, S1, and S3) of AEX and WW ecotypes were collected as mentioned above and immediately frozen in liquid nitrogen, preparing for RNA extraction. Total RNA was extracted with RNAprep Pure Plant Plus Kit (TIANGEN, Beijing, China) and treated with RNase-free DNase I (TIANGEN, Beijing, China), following the manufacturer’s guidebooks. RNA concentration and purity were measured using NanoDrop 2000 (Thermo Fisher Scientific, Wilmington, DE). RNA integrity was assessed using the RNA Nano 6000 Assay Kit of the Agilent Bioanalyzer 2100 system (Agilent Technologies, CA, USA). Twelve RNA sequencing samples resulting from two biological replicates were generated using NEBNext® Ultra™ Directional RNA Library Prep Kit for Illumina® (NEB, Ipswich, MA, USA) following the manufacturer’s recommendations. Library quality was assessed on the Agilent Bioanalyzer 2100 system. After cluster generation, the library preparations were sequenced on an Illumina Hiseq Xten platform (Illumina Inc., San Diego, CA, USA) and paired-end reads were generated. The raw sequencing files of transcriptomic data are now available in NCBI SRA database with accession number PRJNA855119.

### Quality control, assembly, and gene annotation

Clean data were obtained by the software fastp with default parameter, removing reads containing adapter, poly-N, and low-quality reads from raw data. The clean data with high quality of the three treatments across two ecotypes were subsequently *de novo* assembled into a joint transcript using Trinity v2.13.0 with default parameter. To provide insights into the functions of newly identified *A. squarrosum* genes, annotation for all unigenes was performed by running BLASTN against the following databases: NR (NCBI non-redundant protein sequences database); Pfam (The database of Homologous protein family); COG (The database of Clusters of Protein homology); Swiss-Prot (A manually annotated non-redundant protein sequence database); KOG (The database of Clusters of protein homology); KEGG (The database of Kyoto Encyclopedia of Genes and Genomes, [90]); GO (Gene Ontology database) with an E-value threshold of 1e-5 as significant hits.

### Identification and biological function analysis of drought memory genes (DMGs)

Read counts of each sample were standardized by transferring into FPKM (Fragments per Kilobase Million) following the formula FPKM = total exon reads / (mapped reads (Millions) × exon length(kb)), then PCA and Pearson’s correlation coefficient of the were calculated based on FPKM of each sample and visualized by ggplot2 in R software (v4.0.5) to evaluate the repeatability of biological duplicates and differences between samples. Differential expression analysis across R0, S1, and S3 was performed using the DESeq2 R package (1.10.1). Genes met an adjusted P-value < 0.05 and the absolute value of log_2_(Fold change) ≥ 1 was assigned as DEG. As described in Ding Y. 2013, these genes could be further sorted into 4 subgroups of DMGs with differentially expression profiles in S1 versus S3, designated as [+/+], [−/−], [+/−], and [−/+]; and 2 subgroups of non-memory genes with a similar transcriptional level in S1 and S3, denoted as [+/=] and [−/=]. Additionally, those genes with no obvious change during R0 versus S1 but deferentially expressed in S3 formed the ‘late-responsive gene’ category and were named as [=/+] or [=/−] [15]. To find commonality and characteristic in drought memory between different species, DMGs identified in AEX and WW (regarded as two independent species) were subjected to orthologue seeking by the software OrthoFinder with default parameter against DMG sets of three plants reported in previous researches [13, 15, 16]. Based on the result generated from the five species (AEX, WW, *A. thaliana*, *Z. mays*, and *P. virgatum*), all the orthologue groups (OGs) were divided into three sets: OGs embraced in at least one of the three other species (labeled “Other plants”), OGs embraced in AEX, and in WW (labeled “AEX” and “WW”, respectively). Veen diagram was then drawn for the three OG sets. Gene Ontology (GO) and KEGG enrichment analysis of DMGs was implemented by the clusterProfiler R package, using hypergeometric testing to find GO and KEGG entries that are significantly enriched compared to the in-house genome background. Protein sequences of all the DMG identified in *A. squarrosum* were submitted to KEGG Automatic Annotation Server (KAAS, https://www.genome.jp/tools/kaas/). According to the result of Bidirectional Best Hit (BBH) against KAAS database, metabolic network of DMG was visualized manually by Microsoft PowerPoint.

### Co-expression analysis and protein-protein interaction network

The identification of transcription factors (TFs) and transcription regulators (TRs) in DMG was conducted by submitting protein sequences in TFDB online server (http://planttfdb.gao-lab.org/). To figure out the regulatory pattern of drought memory TFs, DMGs within each hierarchy (i.e., plants shared DMGs, AS DMGs, AEX DMGs, and WW DMGs) were firstly divided into regulatory DMGs, namely TFs and TRs, and functional DMGs, e.g., enzyme-coding genes. Then, co-expression analysis between the two aforementioned categories was conducted by R 4.0.5, based on the Pearson’s correlation coefficient. A heatmap of co-expression was generated by TBtools based on the correlation coefficient > 0.9, with p-values less than 0.05. Furthermore, amino acid sequences of candidate functional DMGs were also submitted into String v11.5 (https://www.string-db.org/) with default parameters to find probable protein-protein interactions (PPI). PPI network was visualized by Cytoscape v3.9.0, hub gene mining was conducted via a Cytoscape build-in plugin, cytoHubba.

### Q-PCR validation of the NGS data

In our previous study, *AsACTIN-7*, the universal reference gene was found unstable under the recurring drought treatment; Thus, the optimal reference gene selection was conducted and the combination of *AsUBC22* + *AsPP2A* was determined as the best reference for *A. squarrosum* under repeating water deficit (data unpublished). The transcriptome data in the present research were validated by real-time fluorescence quantitative PCR (q-PCR). The relatively expression of 10 randomly chosen TF switches (5 of each ecotype) were calculated by 2^−ΔΔCt^ method, with 3 biological replicates and 3 technique repeats. *AsUBC22* + *AsPP2A* was used as the internal reference. Primer sequences of the reference genes and examined genes were listed in Additional File 14.

**Table 1:** Statistics of DMG in *A. squarrosum* and previously studied species

Species	*A. squarrosum*		*P. virgatum*	*Z. mays*	*A. thaliana*
	AEX_ecotype	WW_ecotype			
**Total genes by RNA-seq**	**43,170**	**41,458**	**47,207**	**39,635**	**33,555**
**Drought response (DEG)**	**5,379(12.5%)**	**4,436(10.7%)**	**1,566(3.3%)**	**2,062(5.2%)**	**6,579(19.6%)**
Induced	2,891	1,881	811	1,636	3,396
Repressed	2,848	2,555	755	426	3,183
**Non-memory genes**	**3,603(67.0%)**	**3,052(68.8%)**	**825(52.7%)**	**1,246(60.4%)**	**4,616(70.2%)**
Induced [+/=] R0 < S1 = S3	1,893	1,211	337	941	2,177
Repressed [−/=] R0 > S1 = S3	2,070	1,841	488	305	2,439
**Memory genes**	**1,776(33.0%)**	**1,384(31.2%)**	**741(47.3%)**	**816(39.6%)**	**1,963(29.8%)**
[+/+] R0 < S1 < S3	138(7.8%)	118(8.5%)	93(12.6%)	162(19.9%)	362(18.4%)
[−/−] R0 > S1 > S3	128(7.2%)	166(12.0%)	113(15.2%)	72(8.8%)	310(15.8%)
[+/−] R0 < S1 > S3	860(48.4%)	552(39.9%)	381(51.4%)	533(65.3%)	857(43.7%)
[−/+] R0 > S1 < S3	650(36.6%)	548(39.6%)	154(20.8%)	49(6.0%)	434(22.1%)
**Late-response genes**	**1,344(3.1%)**	**1,058(2.6%)**	**5,639(11.9%)**	**2,924(7.4%)**	**1,371(4.1%)**
[=/+] R0 = S1 < S3	756	606	3,158	1,678	798
[=/−] R0 = S1 > S3	588	452	2,860	1,246	573
**Reference**	The present research		[13]	[16]	[15]

Note: The percentages of non-memory genes and drought memory genes were their proportion in drought response genes, while the percentage of late-response genes was their proportion in total genes identified by RNA-seq.

## Electronic supplementary material

Below is the link to the electronic supplementary material.

Supplementary Material 1

Supplementary Material 2

Supplementary Material 3

Supplementary Material 4

Supplementary Material 5

Supplementary Material 6

Supplementary Material 7

Supplementary Material 8

Supplementary Material 9

Supplementary Material 10

Supplementary Material 11

Supplementary Material 12

Supplementary Material 13

Supplementary Material 14

## Data Availability

The datasets generated and/or analyzed during the current study are available in the NCBI SRA repository, with accession number PRJNA855119 (https://www.ncbi.nlm.nih.gov/bioproject/PRJNA855119). All data generated or analyzed during this study are included in this published article and its supplementary information files.

## Abbreviations

DEG: Differentially expressed gene
DMG: Drought memory gene
OG: Orthologue groups
PPI: Protein-protein interaction
RWC: Relatively water content
SMC: Soil moisture content
TF: Transcription factor
