# Supplementary figures and images for: A novel TF molecular switch-mechanism found in two contrasting ecotypes of a psammophyte, Agriophyllum squarrosum, in regulating transcriptional drought memory
Source: BMC Plant Biol. 2023 Mar 30;23:167. doi: 10.1186/s12870-023-04154-6 (PMC10061855; doi:10.1186/s12870-023-04154-6)

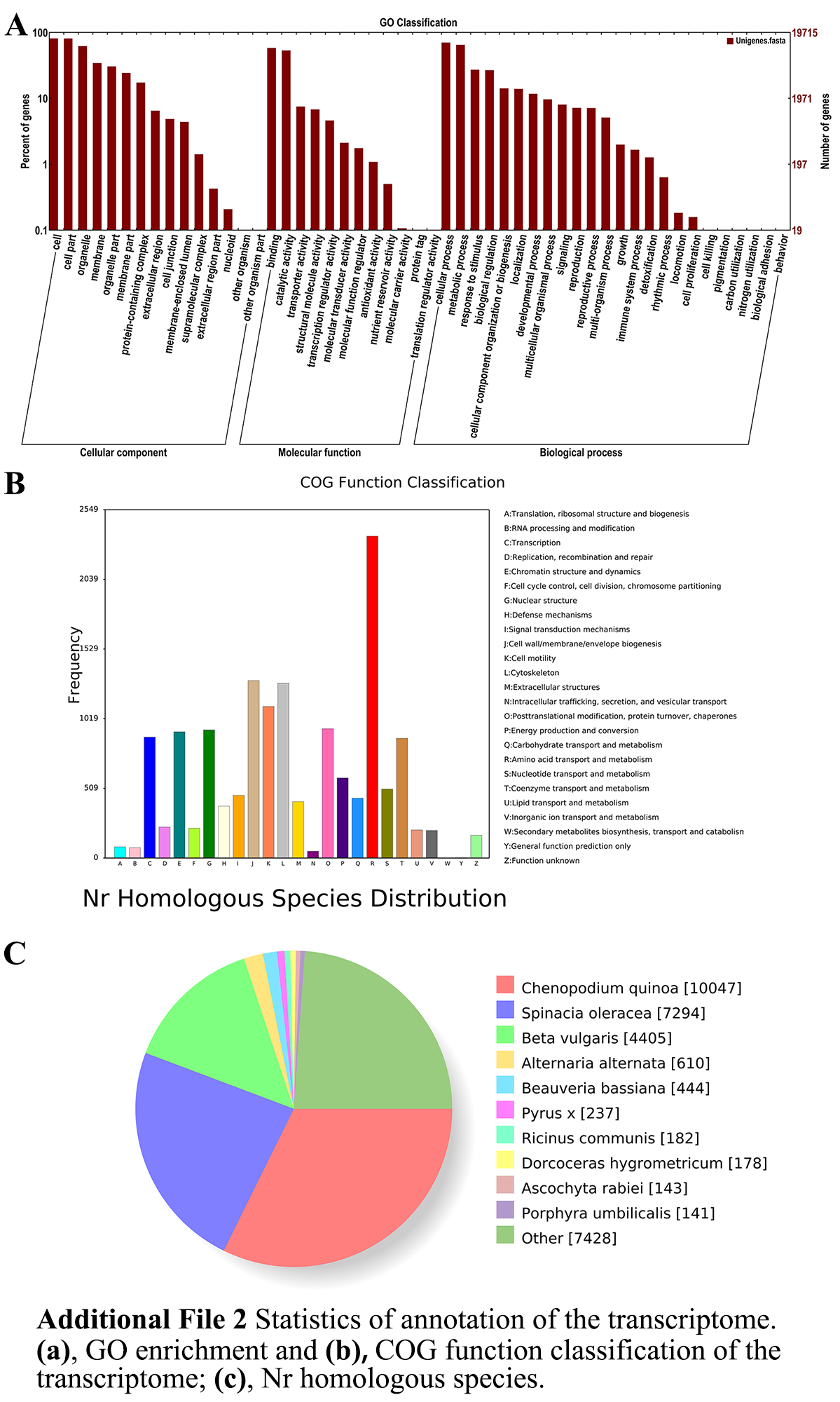

Supplement: Supplementary file 2 — Supplementary Material 2 [file 12870_2023_4154_MOESM2_ESM.tif]

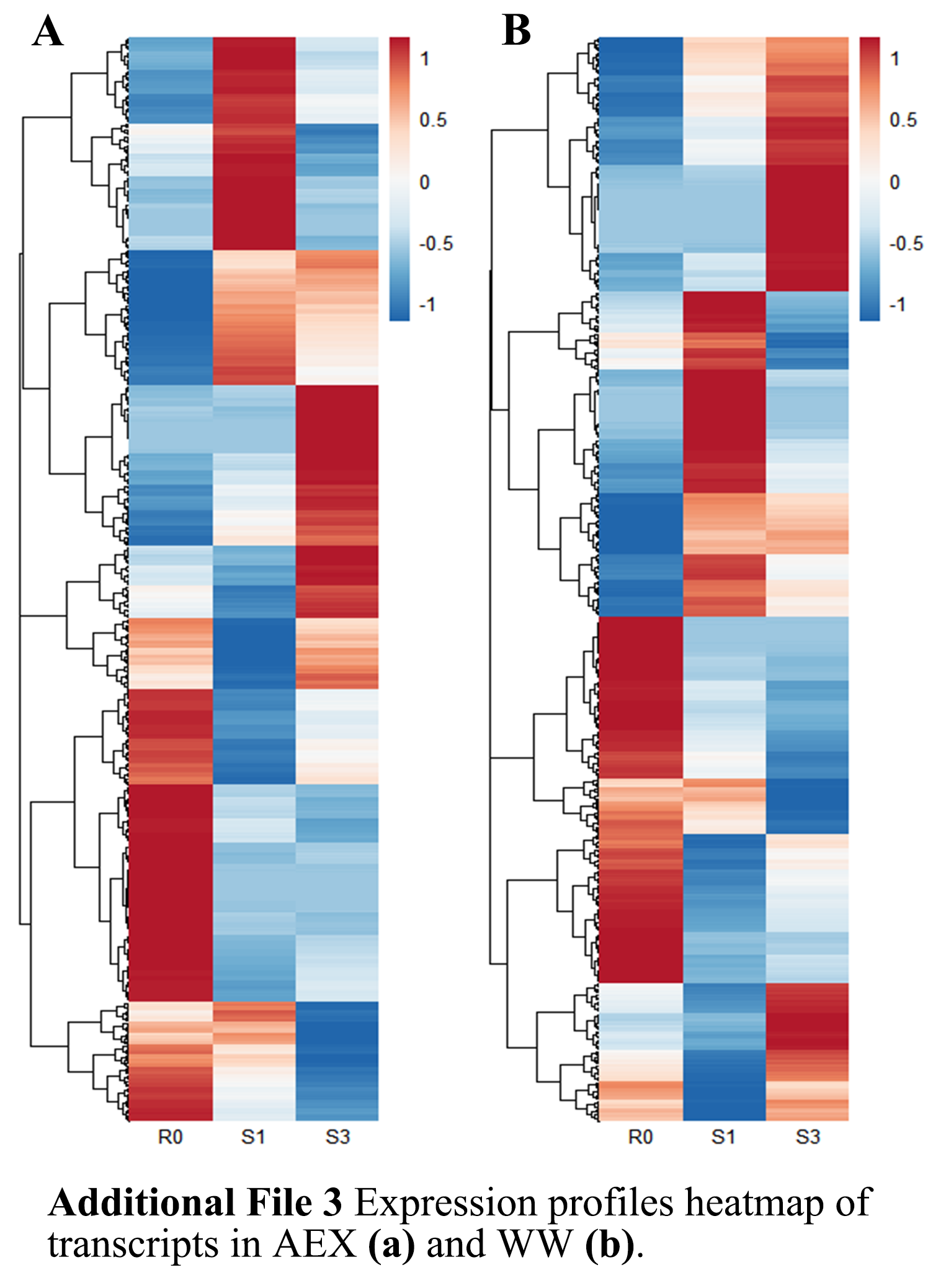

Supplement: Supplementary file 3 — Supplementary Material 3 [file 12870_2023_4154_MOESM3_ESM.tif]

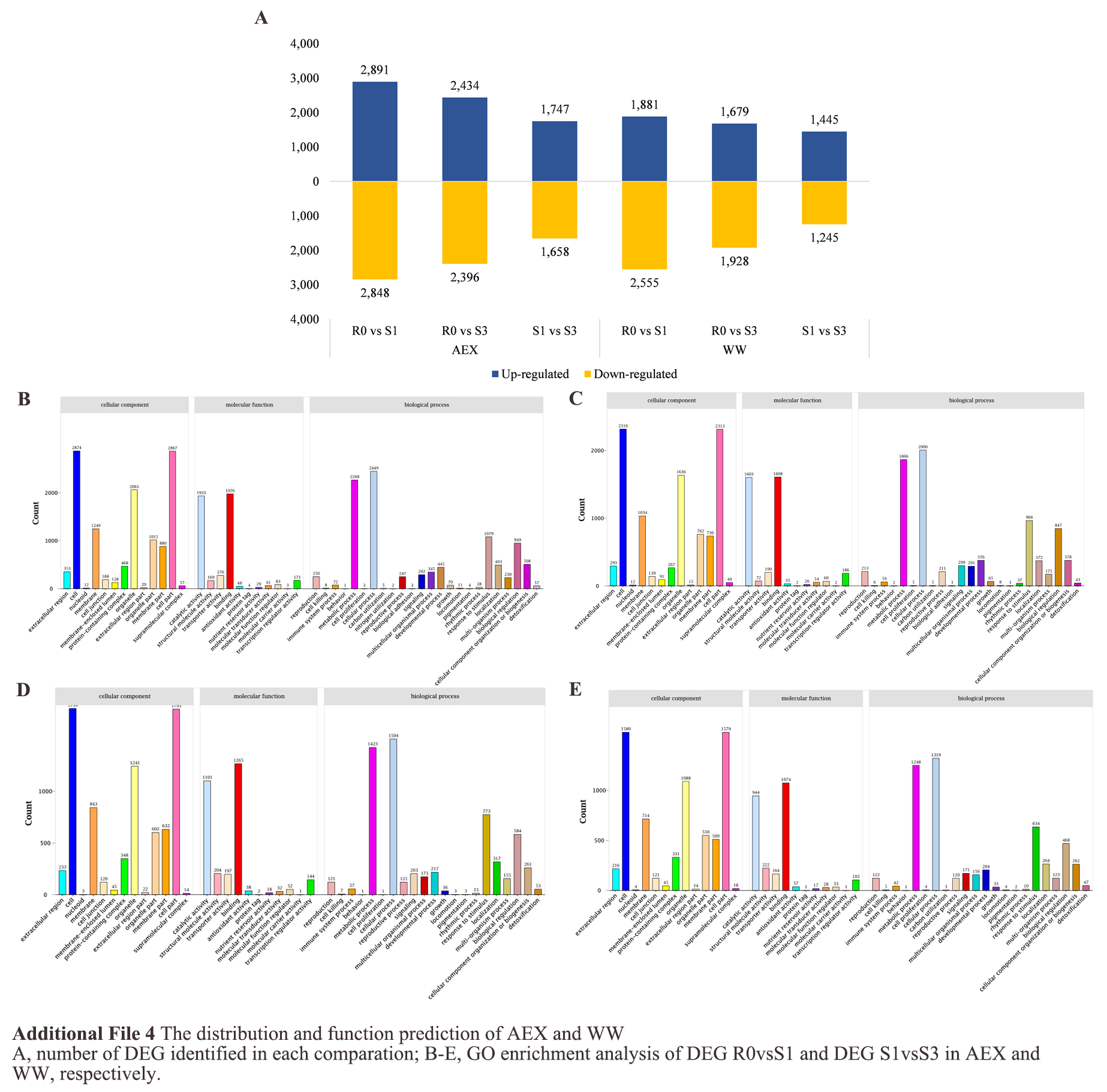

Supplement: Supplementary file 4 — Supplementary Material 4 [file 12870_2023_4154_MOESM4_ESM.tif]

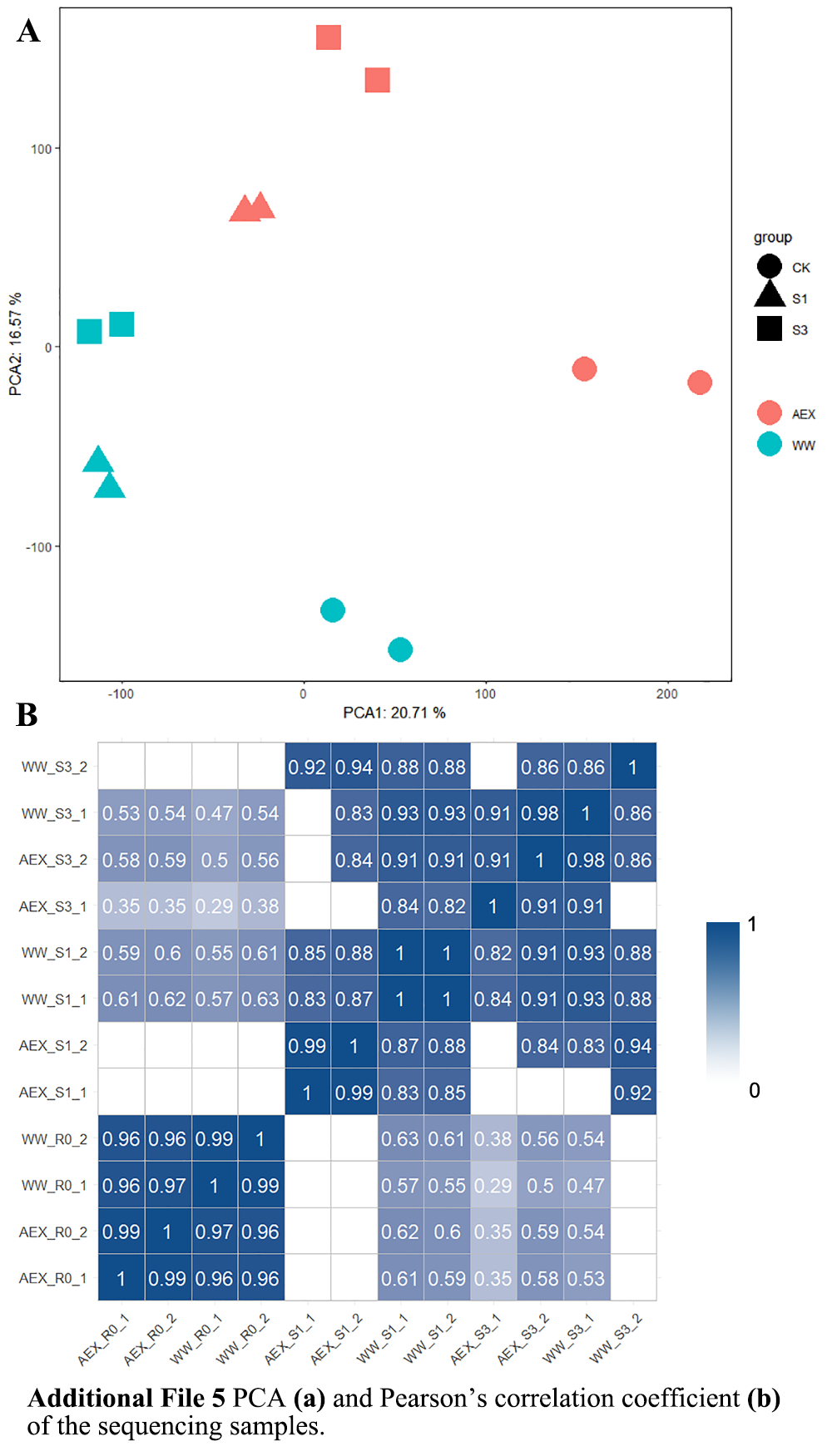

Supplement: Supplementary file 5 — Supplementary Material 5 [file 12870_2023_4154_MOESM5_ESM.tiff]

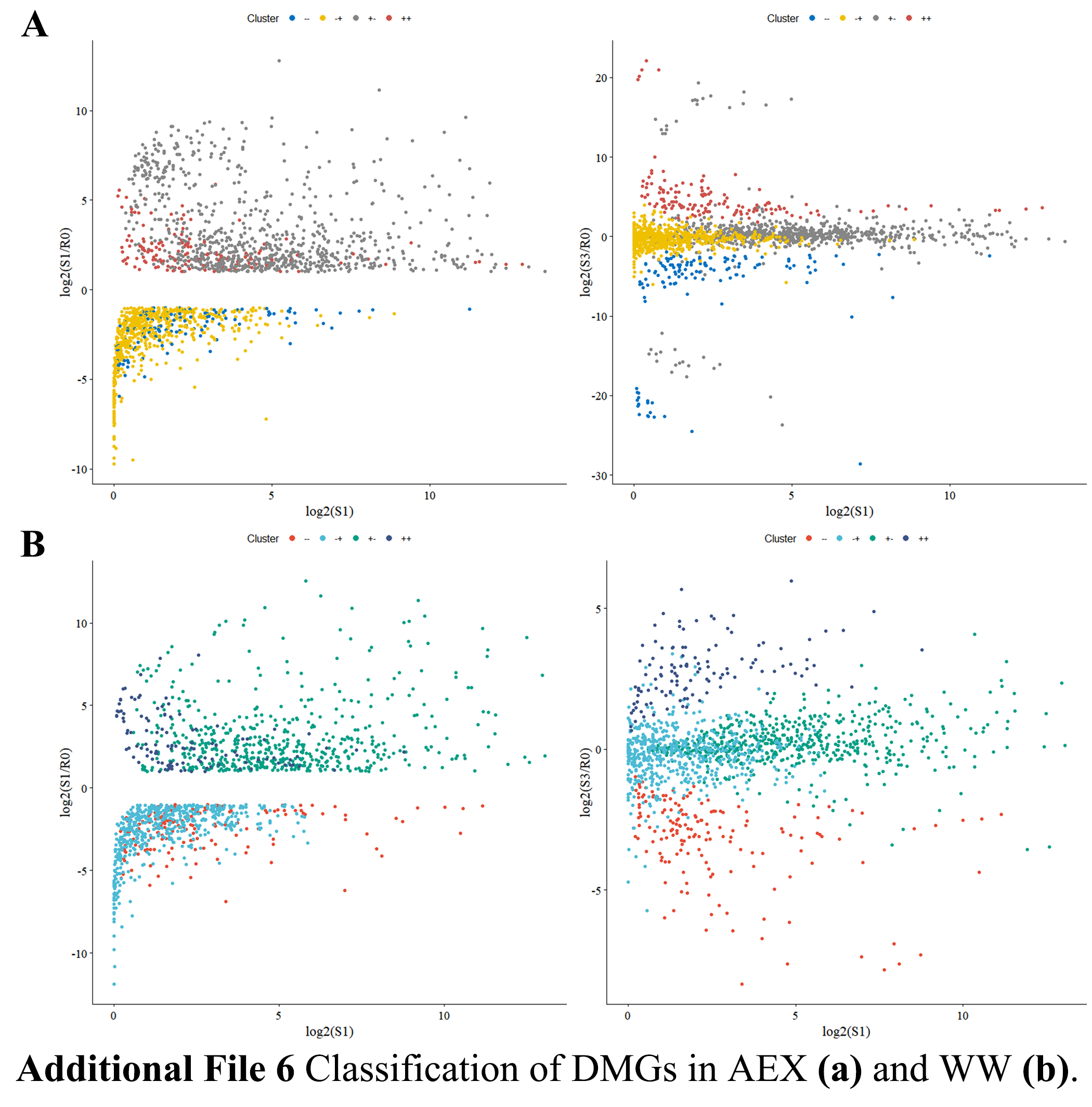

Supplement: Supplementary file 6 — Supplementary Material 6 [file 12870_2023_4154_MOESM6_ESM.tif]

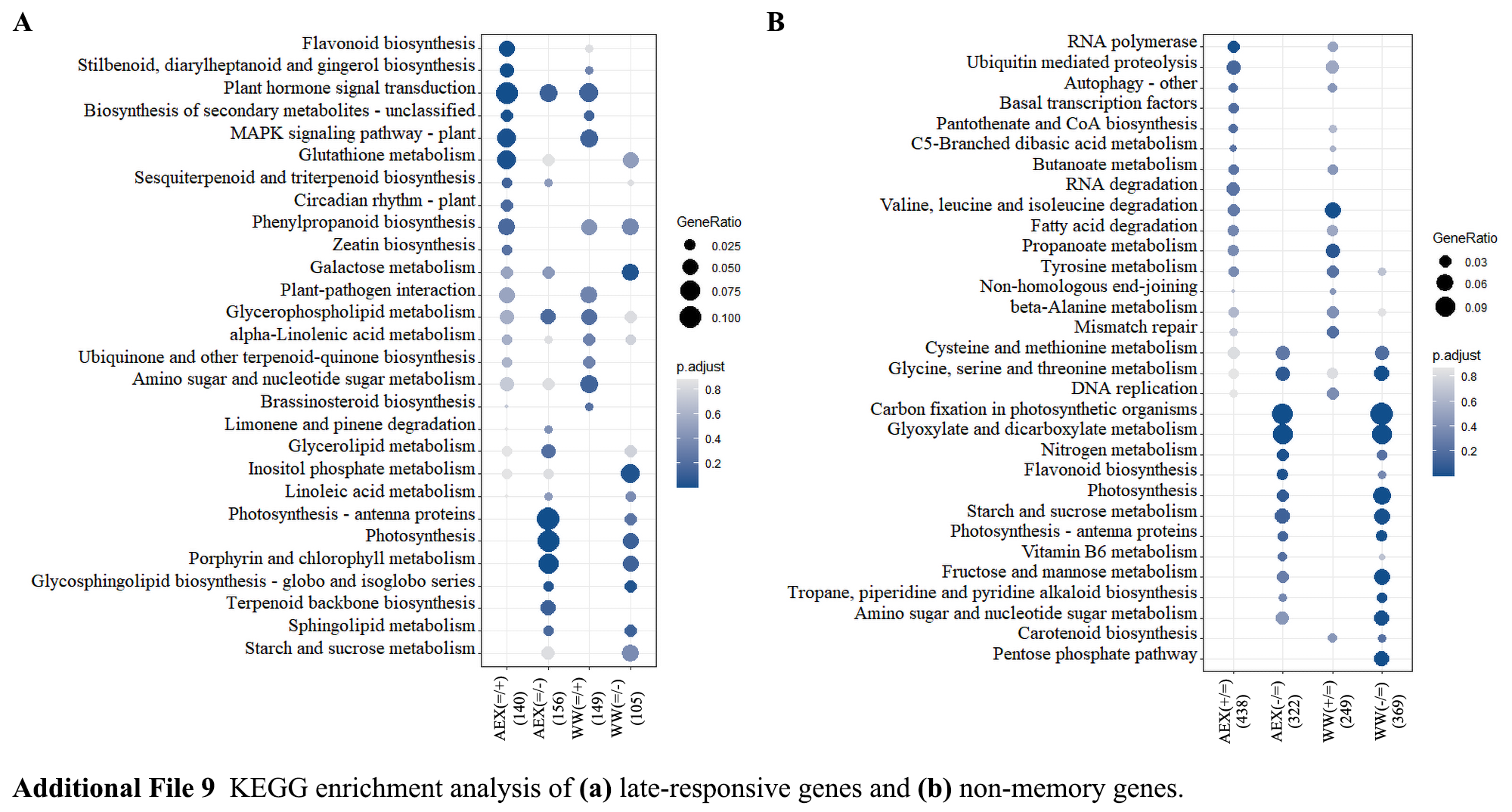

Supplement: Supplementary file 9 — Supplementary Material 9 [file 12870_2023_4154_MOESM9_ESM.tiff]

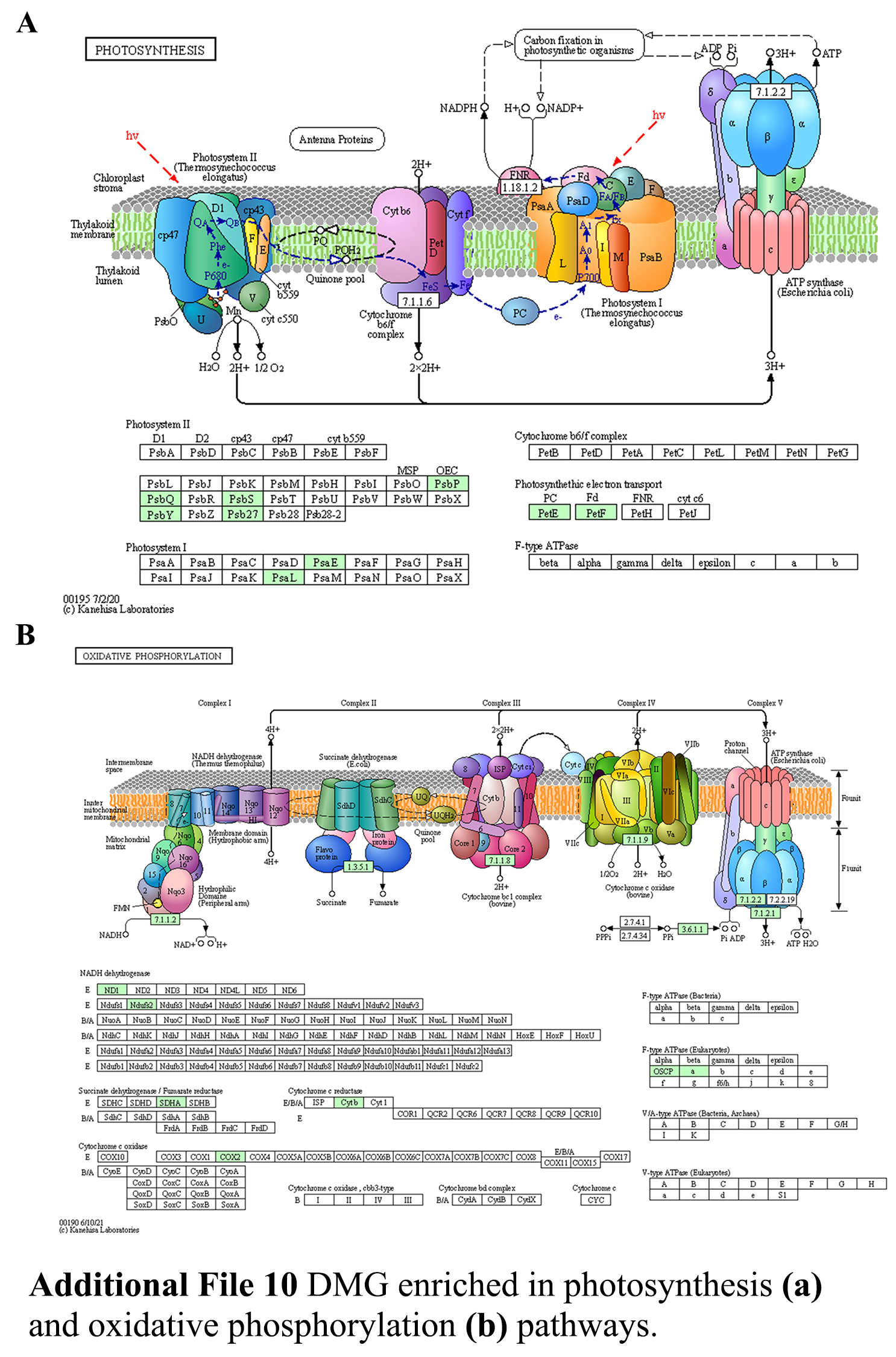

Supplement: Supplementary file 10 — Supplementary Material 10 [file 12870_2023_4154_MOESM10_ESM.tif]

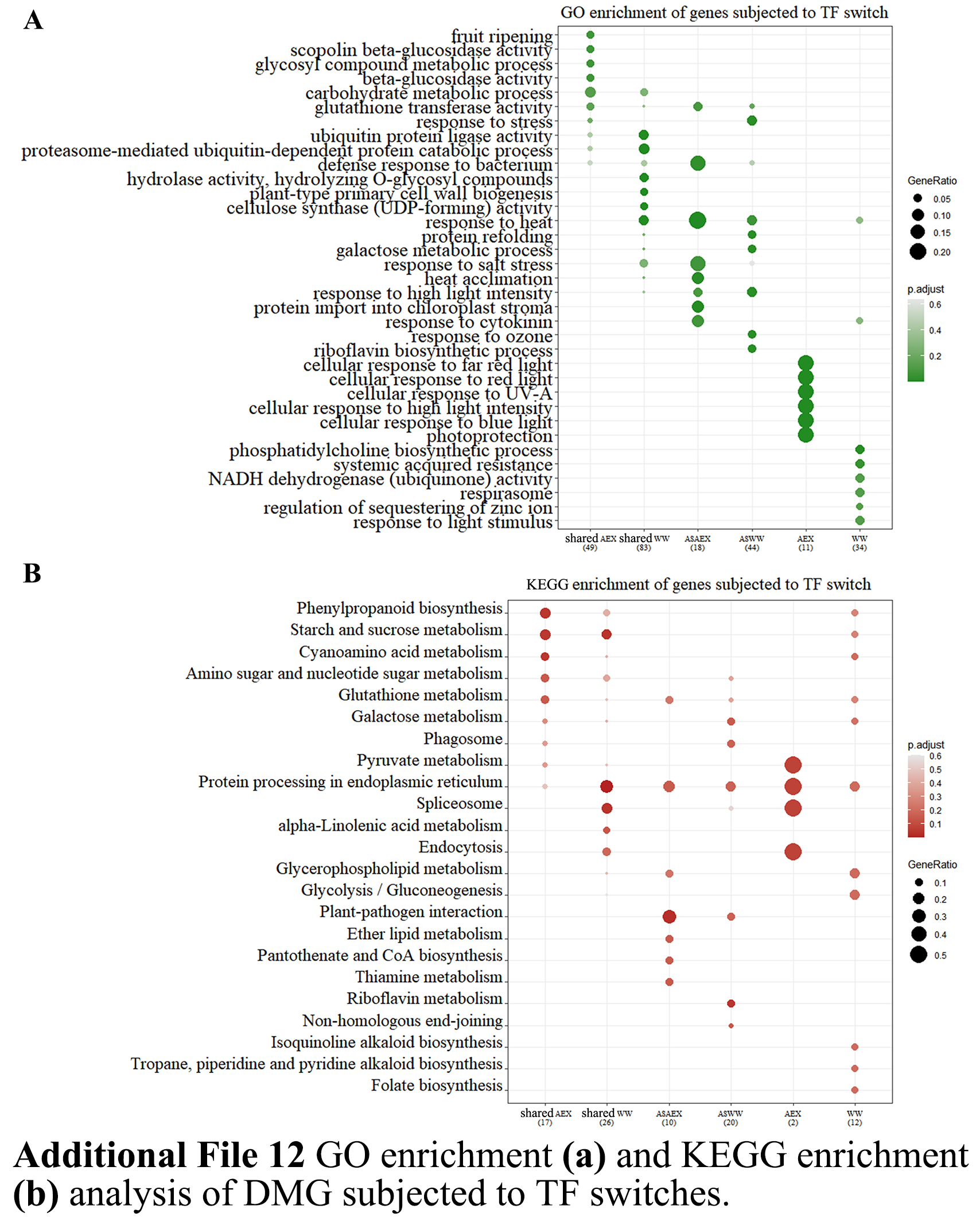

Supplement: Supplementary file 12 — Supplementary Material 12 [file 12870_2023_4154_MOESM12_ESM.tif]
